# Bayesian Top-Down Protein Sequence Alignment with Inferred Position-Specific Gap Penalties

**DOI:** 10.1371/journal.pcbi.1004936

**Published:** 2016-05-18

**Authors:** Andrew F. Neuwald, Stephen F. Altschul

**Affiliations:** 1 Institute for Genome Sciences and Department of Biochemistry & Molecular Biology, University of Maryland School of Medicine, Baltimore, Maryland, United States of America; 2 National Center for Biotechnology Information, National Library of Medicine, National Institutes of Health, Bethesda, Maryland, United States of America; University College London, UNITED KINGDOM

## Abstract

We describe a Bayesian Markov chain Monte Carlo (MCMC) sampler for protein multiple sequence alignment (MSA) that, as implemented in the program GISMO and applied to large numbers of diverse sequences, is more accurate than the popular MSA programs MUSCLE, MAFFT, Clustal-Ω and Kalign. Features of GISMO central to its performance are: **(i)** It employs a “top-down” strategy with a favorable asymptotic time complexity that first identifies regions generally shared by all the input sequences, and then realigns closely related subgroups in tandem. **(ii)** It infers position-specific gap penalties that favor insertions or deletions (indels) within each sequence at alignment positions in which indels are invoked in other sequences. This favors the placement of insertions between conserved blocks, which can be understood as making up the proteins’ structural core. **(iii)** It uses a Bayesian statistical measure of alignment quality based on the minimum description length principle and on Dirichlet mixture priors. Consequently, GISMO aligns sequence regions only when statistically justified. This is unlike methods based on the *ad hoc*, but widely used, sum-of-the-pairs scoring system, which will align random sequences. **(iv)** It defines a system for exploring alignment space that provides natural avenues for further experimentation through the development of new sampling strategies for more efficiently escaping from suboptimal traps. GISMO’s superior performance is illustrated using 408 protein sets containing, on average, 235 sequences. These sets correspond to NCBI Conserved Domain Database alignments, which have been manually curated in the light of available crystal structures, and thus provide a means to assess alignment accuracy. GISMO fills a different niche than other MSA programs, namely identifying and aligning a conserved domain present within a large, diverse set of full length sequences. The GISMO program is available at http://gismo.igs.umaryland.edu/.

## Author Summary

Existing multiple alignment programs typically utilize (i) bottom-up progressive strategies, which require the time-consuming alignment of each pair of input sequences, (ii) *ad hoc* measures of alignment quality, and (iii) pre-specified, uniformly-defined gap penalties. Here we describe an alternative strategy that first provisionally aligns regions generally shared by all the input sequences, and then refines this alignment by iteratively realigning correlated sequences in tandem. It infers position-specific gap penalties directly from the evolving alignment. It avoids suboptimal traps by stochastically traversing the complex, correlated space of alignments using a statistically rigorous measure of alignment quality. For large sequence sets, this approach offers clear advantages in alignment accuracy over the most popular programs currently available.

This is a *PLOS Computational Biology* Methods paper.

## Introduction

A common starting point for the computational analysis of proteins is the construction of a multiple sequence alignment (MSA). Insofar as they result from protein functional similarities and differences, the patterns of residue conservation and divergence within such an alignment provide clues to biological function. Of course the biological relevance of any observed patterns depends upon an alignment’s accuracy, and alignments of larger sequence sets have greater statistical power. For biologically appropriate scoring systems applied to more than a very small number of sequences, however, no optimization procedures are known that are both tractable and rigorous; thus all practical MSA programs rely upon heuristic methods.

The most widely used general approach to multiple alignment is the progressive technique [1], which constructs an MSA by combining sub-alignments, beginning with similar pairs and progressing to more distantly related groups. Many progressive alignment programs are slowed by the need to construct a “guide tree”, which specifies the order in which sequence subgroups are aligned, from pairwise alignment scores. For *n* sequences of a fixed average length, it requires O(*n*^2^) time to compute such scores, and this becomes the time-dominating step for large *n*. One way around this problem is to iteratively refine a guide tree and MSA starting from an initial crude guide tree, an approach used in the popular MUSCLE [2] and MAFFT [3–5] programs. The MAFFT PartTree [6] and Clustal-Ω mBed [7] algorithms yield guide tree construction times of *n*log *n*. A recent O(*n*) approach [8] is to use a simple chained guide tree, and add individual sequences to a growing alignment in an arbitrary order.

An alternative O(*n*) method that avoids aligning all sequences to one another is to use Markov chain Monte Carlo (MCMC) sampling to iteratively align sequences to an evolving hidden Markov model (HMM) [9–11]. Our approach initially uses a block-based HMM to represent islands of similarity within otherwise dissimilar sequences. The number of the blocks and their lengths are first sampled randomly from a prior distribution, and placed randomly but co-linearly within each sequence. Posterior HMM parameters are derived from this alignment. Next, an arbitrary sequence *S* is removed from the alignment, and the model’s parameters are updated and then used to sample new locations for its blocks within *S*. This process may be iterated an arbitrary number of times. Also, both blocks and columns at the edges of each block are iteratively sampled in or out of the alignment and of the corresponding model. Sampling continues in this way until the log-likelihood ratio (LLR) implied for the evolving HMM fails to improve over a specified number of iterations. All alignments are sampled using probabilities proportional to their LLRs. Note that the hmmt program iteratively refines an HMM in a similar manner [12].

Aligning distantly related sequences presents major algorithmic and statistical challenges because such sequences typically share similarity only within a common structural core, with sizable insertions often occurring between core elements. Classical dynamic programming alignment algorithms typically have difficulty spanning these insert regions because the log-odds scores associated with weakly conserved core elements are often too low to offset the gap penalties incurred. Fortunately, even when the conserved blocks are very subtle, an MCMC strategy can take advantage of a large number of input sequences to detect weak yet statistically significant similarities.

Two factors have tended to slow previous MCMC sampling procedures, or to trap them in local optima. The first is the inclusion of correlated sequences within the input. When a set of such sequences is misaligned to the main body of sequences, it favors recurring misalignment when individual sequences from the set are resampled. This problem may be partially addressed by removing from the program’s input all but one sequence among closely related sets; these sequences may be added to the alignment at the program’s end. The second factor is the difficulty in accurately identifying the number and locations of aligned columns corresponding to the structural core, and the corresponding placement of indels. A previous sampler addressed this problem with only partial success by splitting or joining contiguous blocks, extending or trimming blocks, and by allowing short indels within a block [10].

A critical issue for multiple alignment programs is how they internally assess sequence alignment quality. One widely used measure is the sum of the implied pairwise scores, but this measure lacks a good mathematical justification. Previous MCMC programs introduced measures with a rigorous statistical basis by sampling over the posterior probability distribution defined by a statistical model for aligned columns [9, 13]. However, they employed uniform HMM transition probabilities (i.e., gap penalties) [10], which fail to model position-specific indels with comparable generality.

In this article, we describe a new approach to MSA, whose main features are as follows. **(a)** It uses a **top-down strategy** and MCMC sampling to align *n* sequences of a fixed average length in O(*n*) time. It achieves this by first globally aligning input sequences to a block-based model, then generalizing this model by converting aligned blocks into a continuous, gapped alignment and refining it by repeated Markov chain resampling. **(b)** It employs a Bayesian generative statistical model and the minimum description length (MDL) principle [14] to measure the quality of alignments, and seeks to optimize this measure. **(c)** It employs Dirichlet mixture priors [15, 16] constructed using recently described optimization procedures [17]. **(d)** It dynamically infers HMM position-specific transition probabilities (i.e., gap penalties) based on the evolving alignment. **(e)** It uses new sampling strategies for correlated sequences to efficiently escape from local optima. We have implemented this approach in a program called GISMO (Gibbs Sampler for Multi-alignment Optimization) and demonstrate here that it can align large numbers of diverse protein sequences on average more accurately than existing methods.

### The GISMO sampler

GISMO shares certain algorithmic and statistical features with an earlier version of this sampler [10], including the optimization of a hidden Markov model (HMM); the corresponding statistical model is reviewed in Methods. In this section we describe features key to our new sampler.

### Top-down alignment strategy

**Fig 1:**
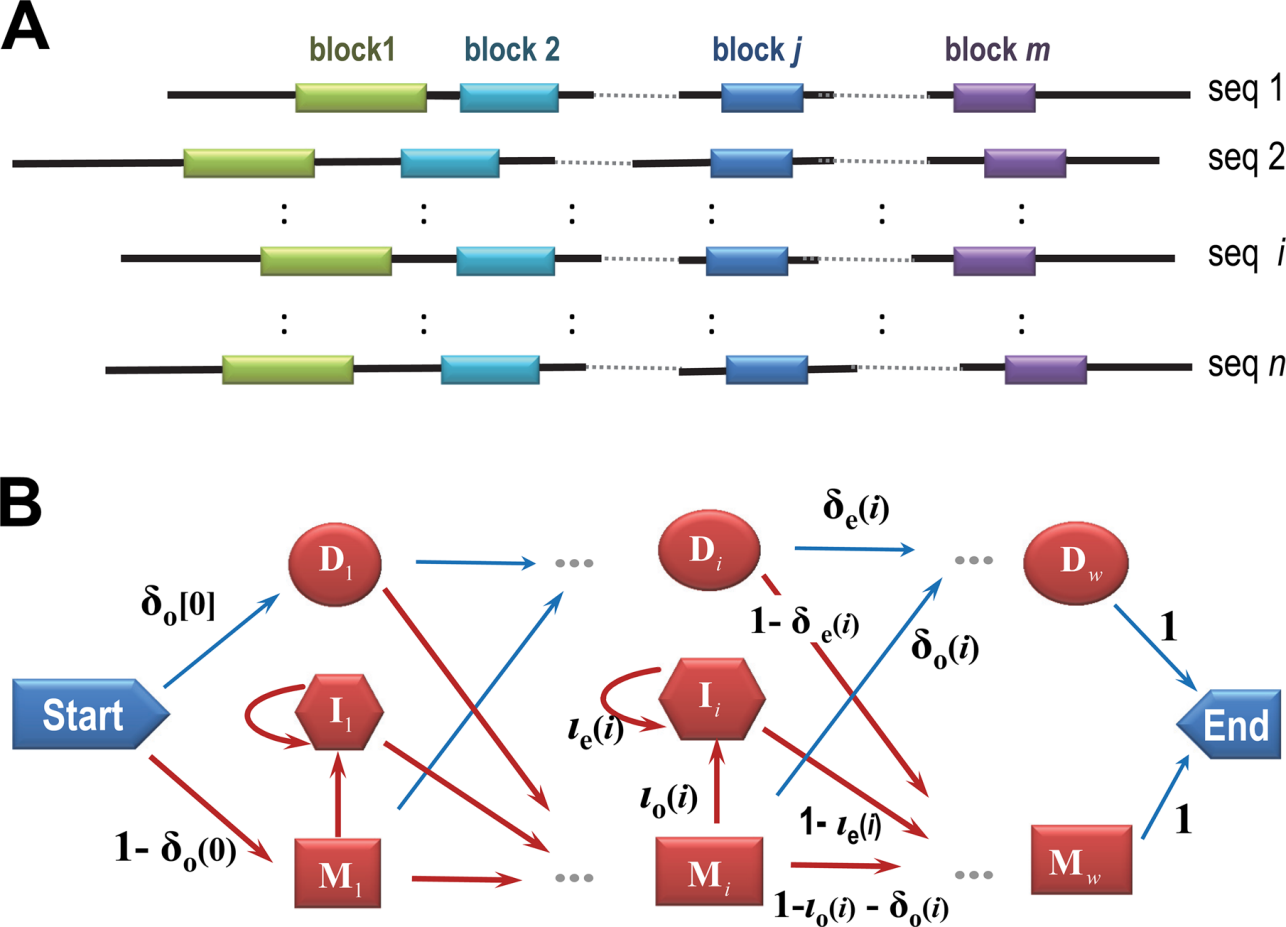
GISMO block-based and hidden Markov models. **A**. Schematic of a hypothetical GISMO phase 1 block based alignment, which is initialized to consist of many short, ungapped aligned blocks. **B**. Architecture for the GISMO phase 2 HMM. Red transition arrows between states emit residues. Transition probabilities are inferred from the sequence data. Note that the HMM is local with respect to each sequence but global with respect to the model.

Most multiple alignment methods utilize a “bottom-up” progressive alignment strategy. That is, they start by aligning the most closely-related sequences and progressing to those more distantly related. GISMO takes an inverted, “top-down” MCMC approach that starts by aligning, among all sequences, the core regions they share. It first generates a random alignment consisting of many short (5- to 15-column) co-linear aligned blocks (**Fig 1A**). It then samples sequences, columns and blocks into and out of this alignment, proportionally to their likelihoods as implied by the underlying statistical model. The resulting relatively crude block-based alignment is then converted into a single HMM (**Fig 1B**) with position-specific transition probabilities that are allowed to evolve as the sampler progresses (see below). Finally, GISMO applies various sampling strategies to optimize the number of columns and the locations of indels and to realign clusters of correlated sequences that, if sampled individually, could trap the sampler in a suboptimal alignment.

### A measure of alignment quality

A program’s measure of multiple alignment quality, either explicit or implicit, plays a vital role in determining the alignments it will produce. GISMO’s measure corresponds to an underlying generative statistical model, specifically an HMM, and, to the extent that it can efficiently explore alignment space, GISMO will converge on an optimal solution (assuming that sequence weights remain the same; see below). GISMO’s statistical model has several features worth noting. (i) To counter redundancy or bias among the input sequences, GISMO down-weights closely correlated sequences [18]. Early on as the alignment evolves so do the sequences’ inferred weights; in the final stage of sampling these weights remain constant. (ii) GISMO models the tendency for amino acid residues to substitute for one another using Dirichlet mixture priors [15, 16], whose calculation has recently been refined [17]. This improves the statistical model’s sensitivity to biological relationships. See Methods for mathematical details.

### Inferred position-specific gap penalties

As described in Methods, GISMO infers HMM transition probabilities at each model position based on the evolving alignment itself. More specifically, the observed numbers of each type of transition, along with specified prior probability distributions for these transitions, imply posterior probabilities for each transition at each position. These correspond to implicit gap penalties, which favor insertions or deletions (indels) within a given sequence that vary in tandem with where indels have been inferred within other sequences. This tends automatically to favor near block-based alignments—a characteristic that the following column-sampling strategy exploits.

### Column sampling strategies

The HMM’s evolving position-specific transition probabilities tend to align conserved regions of the proteins as contiguous blocks, separated by insertions of varying lengths. To improve the alignment, however, it is desirable for the sampler to explore alternative configurations of aligned columns. To determine the proper extent of an implied block, GISMO adds or removes columns based on their **Bayesian Integral Log-odds** (**BILD**) scores [19]. The BILD formalism arises from the **Minimum Description Length** (**MDL**) principle [14], which provides a criterion for choosing among alternative models for describing a set of data. Conceptually, it suggests that the best model, among a set of alternatives, is that which minimizes the description length of the model, plus the maximum-likelihood description length of the data given the model. Note, however, that early in our sampling, we retain columns that, based on their BILD scores, fail marginally to be statistically supported, in order to allow the sampler time to converge on an accurate alignment. GISMO also will move model columns from one side of a set of insertions to the other, if this improves the aggregate BILD score

### Sequence sampling strategies

When individual sequences are realigned to the evolving HMM, they may be sampled (as described in Methods) one at a time, with the HMM parameters recomputed after each sequence is removed from the alignment. However, this approach encounters difficulties when an alignment consists of distinct clusters of more closely related sequences, because a sampled sequence is biased by the remaining sequences of its cluster to realign as before. Sampling all the sequences of a cluster in tandem can overcome this “stickiness”. GISMO does this in two distinct ways. First, for a cluster *C* of extremely closely related sequences whose mutual alignment lacks indels: (i) GISMO constructs a consensus sequence *S* to represent the sequences in *C*, and prealigns *S* to these sequences; (ii) It removes all the sequences of *C* from the general alignment, and adjusts the implied HMM parameters accordingly; (iii) It aligns *S* to the HMM by sampling; (iv) Using *S* as a template, it places the sequences of *C* back into the general alignment.

Alternatively, for a cluster *C* of somewhat more distantly related sequences: (i) GISMO removes all the sequences of *C* from the general alignment and adjusts the implied HMM accordingly; (ii) It realigns the sequences of *C* in turn to the HMM by sampling. GISMO applies this latter realignment procedure not only to sets of sequences clustered by sequence similarity, but also to groups of sequences that share congruent insertions or deletions or that share a non-consensus residue in an otherwise well conserved column (see **S1 Fig**). In all these cases, by sampling ‘sticky’ sequences in tandem, GISMO is able to escape many local traps in alignment space.

Finally, GISMO applies three different coordinated sampling strategies: (i) It realigns sequences using a ‘purged’ set as follows: first, it groups all sequences into closely-related clusters; second, it retains in the alignment only the one sequence from each cluster closest to the cluster’s consensus sequence; third, it realigns by sampling each of the remaining sequences; finally, it resamples into the alignment each of the sequences that were originally excluded. This step resembles another, recently-described MSA strategy [20]. (ii) It removes in tandem the poorest scoring sequences and then resamples them, under the assumption that poor scores may arise from alignment errors. And (iii) it removes in tandem and then resamples randomly chosen sequence subsets.

### Competitive selection strategy

Given the stochastic nature of MCMC sampling, it is advantageous to focus on refining the best alignment among several initial candidate alignments. GISMO does this as follows. (i) It generates a rough block-based alignment for all input sequences, which it uses to construct clusters of closely related sequences, and then selects one sequence from each cluster for further preliminary alignment. (ii) For these sequences, it independently generates a population of block-based alignments, ten by default. (iii) It converts each of these alignments into an HMM alignment and resamples its sequences permitting the introduction of gaps. (iv) It scores each alignment by its similarity to the other alignments (see Methods). The assumption is that the best alignments will share more similarity with other alignments. Moreover, such agreement may indicate, in the absence of structural information, that a more accurate alignment has been found. (v) It further refines the highest scoring alignments, five by default, and then selects the best of these by the same criterion. (vi) It samples back the remaining sequences, performs additional refinement, and returns a final, full alignment.

## Results

The GISMO program was implemented in C++. We tested GISMO on 408 protein sequence sets; these correspond to those domain alignments within version 3.14 of the NCBI Conserved Domain Database (CDD) [21] that contain at least 50 sequences, at least 10 of which share less than 70% identity to each other. (The CDD MSA identifiers are listed in **S1 Charts**.) These MSAs have been manually-curated in the light of available crystal structures and serve here as gold standards, against which GISMO and other programs’ MSAs may be benchmarked. These alignments contain up to 2,399 sequences and contain in aggregate between 3,583 and 3,929,595 residues. This test set focuses on the principle application motivating the development of GISMO, the accurate alignment of a conserved domain shared by a relatively large number of diverse, full-length sequences that, outside of the shared domain, are otherwise unrelated.

### Comparisons with other programs

GISMO was compared to four widely used MSA programs, MUSCLE (v3.8.31) [2], MAFFT (v7.158b) [3–5], Clustal-Ω (v 1.2.0) [22, 23] and Kalign (v2.04) [24, 25] as well as to Dialign (v2.2) [26, 27], which, like GISMO, is designed to align conserved regions in sequences that share local homology but are otherwise unrelated [28]. For all programs, we obtained the latest versions and used the default parameter settings; for MAFFT this involved using the–auto option, which allows the program to choose the best settings.

### Alignment quality

We assess alignment accuracy using SP-scores, with the CDD alignments as benchmarks. In brief, an SP-score (from "Sum of the Pairs") is the proportion of aligned pairs of residues within a benchmark multiple alignment, that are similarly aligned within a test multiple alignment. Note that the term "SP-score", with a related but distinct meaning, frequently describes elsewhere an objective measure of multiple alignment quality, as opposed to a measure of alignment accuracy with reference to a benchmark, its meaning here. Note also that our benchmark CDD alignments leave many residues in many sequences unaligned, and these are ignored in calculating SP-scores, so a program that aligns these residues is neither penalized nor advantaged. In practice, GISMO leaves many of these residues unaligned as well, in contrast to most other multiple alignment programs. To the extent that one is not merely agnostic about these residues’ proper alignment, but believes they should in fact be left unaligned, GISMO's performance is underestimated here.

**Fig 2:**
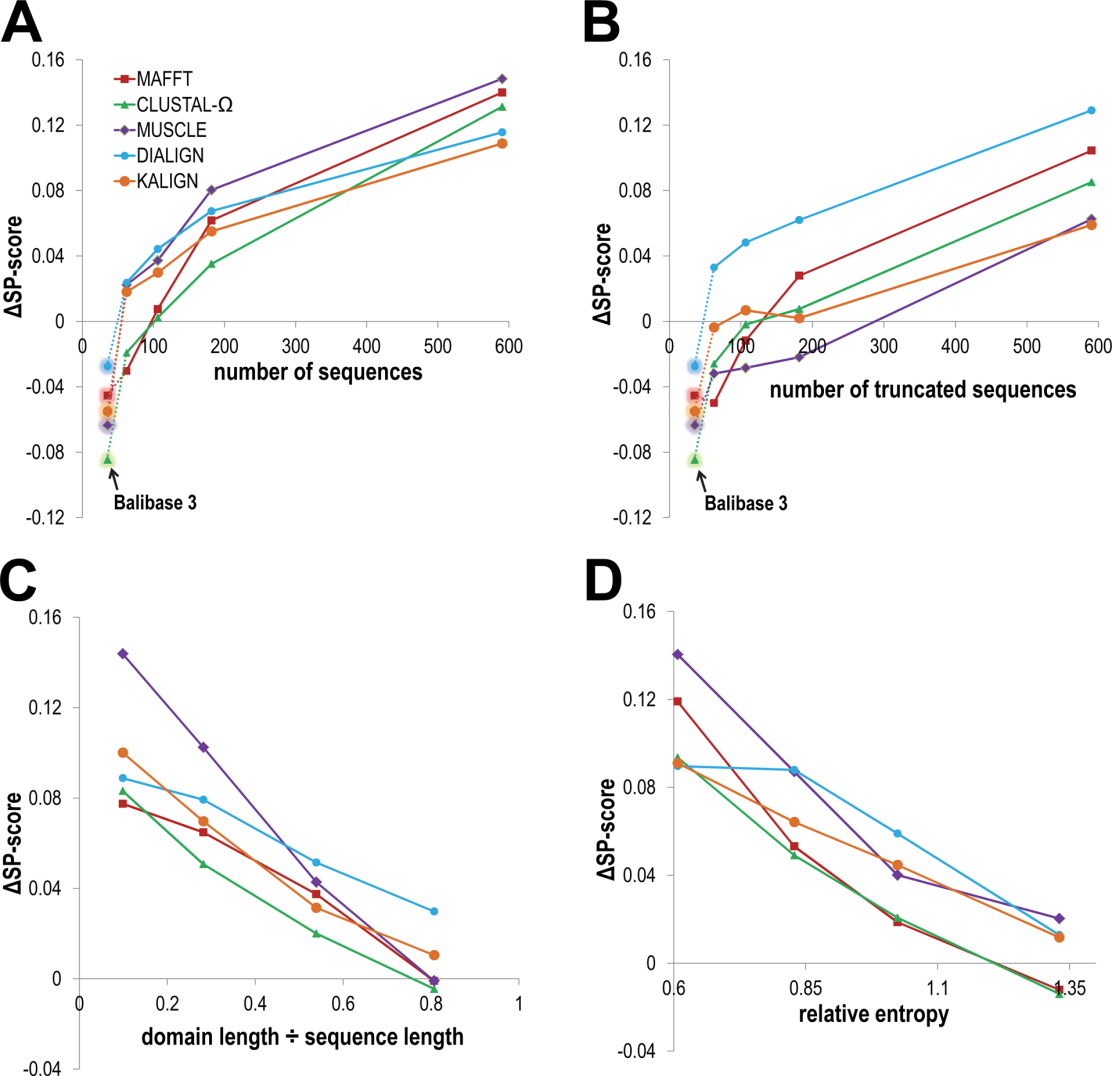
Comparison of GISMO to five other MSA programs. As described in the text, for each analysis the CDD test sets were first ordered based on the property specified on the x-axis and then split into four equal-sized partitions. The x-coordinates for all data points are averages, for the property in question, over the test sets assigned to the various partitions; similarly, the GISMO ΔSP-scores for each program are averages taken over these partitions. **A**. GISMO ΔSP-scores as a function of the number of sequences. For comparison, an additional, leftmost set of data points (shown with back-glow) corresponds to 162 out of 218 Balibase 3 test sets; for the remaining 56 Balibase sets, GISMO failed to find a statistically significant alignment presumably due to sparse data: some of these sets have as few as 4 sequences. **B**.GISMO ΔSP-scores as a function of the number of truncated sequences, as defined in the text. **C**. GISMO ΔSP-scores as a function of the ratio between the domain length and mean sequence length. For sequence sets with low ratios, the shared domain is more challenging to align due to a larger search space. **D**. GISMO ΔSP-scores as a function of average relative entropy (with respect to a standard background amino acid distribution and expressed in nats, with 1 nat = 1/ln(2) bits) over all column positions in each benchmark MSA; sequence diversity can be understood as inversely related to relative entropy. For sequence sets with low relative entropy, the shared domain is more difficult to align due to weaker conservation.

To compare GISMO to other programs, we define the GISMO ∆SP-score as the SP-score for GISMO minus the SP-score for the other program. **Fig 2**plots GISMO ∆SP-scores as a function of several MSA features, namely the number of aligned sequences (**Fig 2A and 2B**), the ratio of domain to mean sequence length (**Fig 2C**), and the relative entropy as an indicator of sequence diversity (**Fig 2D**). The plotted scores are averages for each of four equal-sized partitions of the 408 CDD test sets (i.e., 102 in each partition); the first through fourth partitions contain those test sets having the lowest to highest values, respectively, for the various independent variables. For comparison, **Fig 2A and 2B**also include a fifth partition consisting of 162 Balibase 3 [29] test sets, which contain fewer aligned sequences (35 on average) than do CDD MSAs, are less diverse and are typically truncated versions of the full-length sequences. **Fig 2A** reveals that GISMO performs worse than all of the other programs on the Balibase 3 sequence sets, but progressively better on the progressively larger CDD sequence sets. A plausible explanation for this is that, as the number of sequences increases, so does GISMO’s statistical power to infer subtle sequence properties leading to higher quality alignments. These properties include both residue and indel probabilities at each position in each alignment, with indel probabilities likely to depend on the number of sequences to a greater extent because more observations are required for their accurate estimation. For the 408 CDD MSAs GISMO ∆SP-scores were statistically significant based on a one-tailed Wilcoxon signed rank test [30] with *p* < 10^−5^ for all five programs (see **S1 Statistics**); based on the corresponding Z-scores, CLUSTAL- Ω (Z = +4.31) and MAFFT (Z = +6.02) performed better than MUSCLE, DIALIGN and KALIGN (Z = 8.87, 11.73, and 10.70, respectively).

GISMO’s enhanced alignment quality for larger sequence sets may be due either to improved detection of the conserved domain within full-length sequences or to improved placement of indels within the domain or both. The analyses in **Fig 2B–2D**examine the degree to which GISMO’s superior performance may be due to each of these factors. **Fig 2B** repeats the analysis of **Fig 2A** using truncated versions of the input sequences, which consist of the aligned domain region within each CDD MSA plus ten residues on each side of this region (or as many as exist, if less than ten). On the rightmost partition, containing the largest sets of truncated-sequences, GISMO performs better than the other programs. This indicates that, even when the conserved regions are predefined, GISMO yields more accurate MSAs on sufficiently large data sets. **Fig 2C** plots average GISMO ∆SP-scores as a function of the ratio between conserved domain length and the average sequence length. This ratio corresponds to the relative size of the alignment space over which each MSA program needs to search for the conserved region, and therefore provides a measure of alignment difficulty. On average, GISMO alignment quality relative to these other programs improves as this ratio decreases, that is, as the level of difficulty increases. GISMO’s performance relative to these other programs likewise improves as the level of sequence diversity increases (**Fig 2D**). Together, these analyses suggest that, for large sets of diverse, full-length sequence sets, GISMO is superior both at identifying and aligning conserved domains.

Also worth noting about the analyses in **Fig 2**is MUSCLE’s much improved relative performance on the truncated versus the full-length sets as a function of the number of aligned sequences (compare **Fig 2A and Fig 2B**). This suggests that MUSCLE is better at properly aligning conserved regions than at identifying them within full-length sequences. This also illustrates how each program’s relative performance may be better on some sequence sets and worse on others. In this regard, we surmise that the reliance on benchmark sets with a rather limited range of properties has tended to favor certain MSA program niches over others. In particular, our analysis suggests that programs for aligning large sets of diverse full-length sequences are underrepresented.

### Run-to-run variability

**Fig 3:**
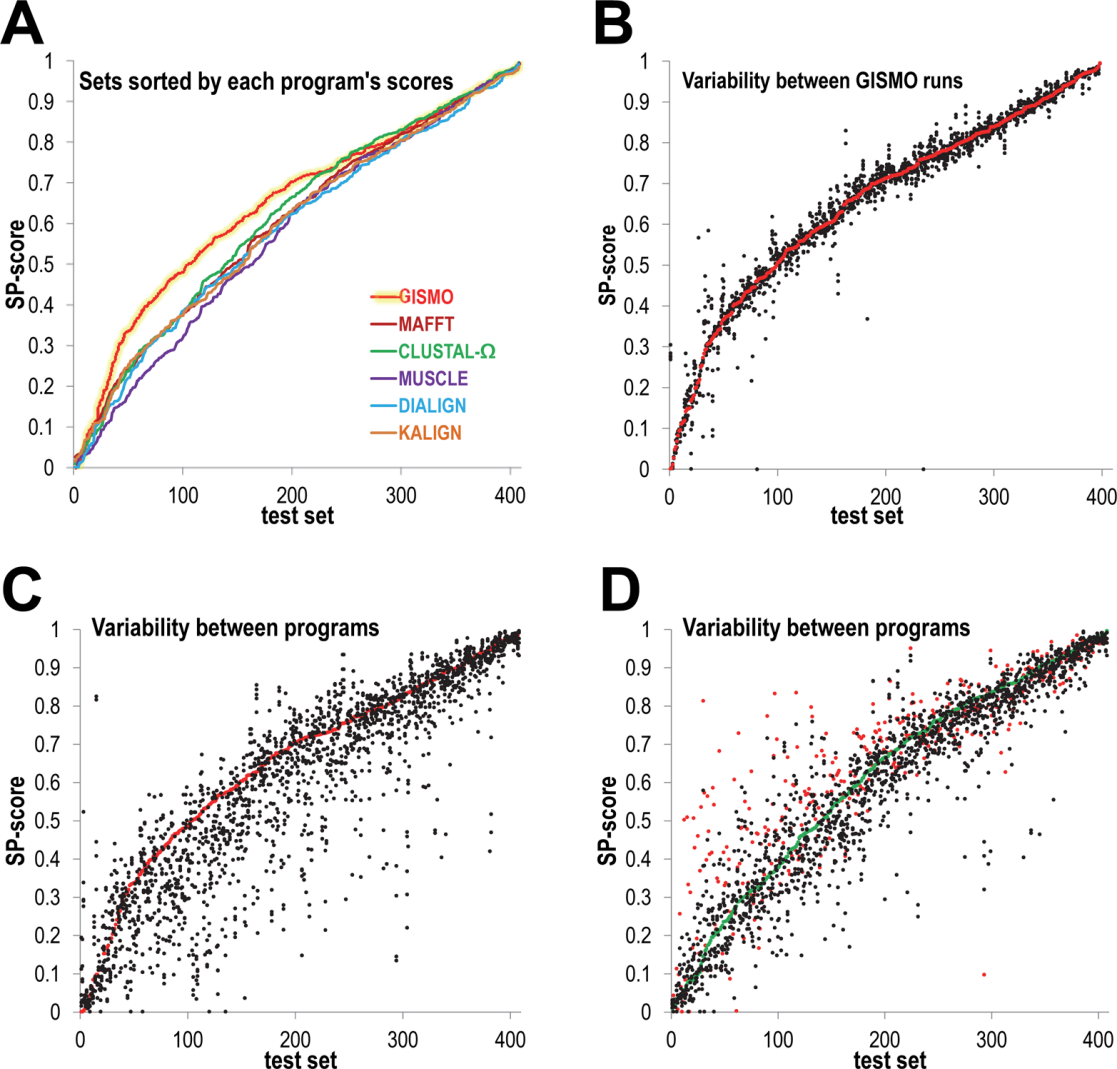
Variability in SP-scores among six GISMO runs and among the six programs GISMO, MAFFT, CLUSTAL-Ω, MUSCLE, Dialign and Kalign. SP-scores are based upon the CDD MSAs as benchmarks and vary from 0 (no correctly aligned sequence pairs) to 1 (all pairs aligned correctly). A. The sorted SP-scores for a single GISMO run (red line with yellow back-glow) compared with the sorted scores for the five other programs. B. Run-to-run variability in SP-scores over six GISMO runs. Test set data points are sorted along the x-axis by the SP-score obtained for each set on the first run (red data points) of six. C. SP-scores for the six programs analyzed, sorted by the GISMO score on each test set. GISMO SP-scores (for a single run) are shown in red. Each red data point and the five black data points (one point for each program) plotted in the same column correspond to the same test set. D. SP-scores for the six programs, sorted by the CLUSTAL-Ω score on each test set. Data points for GISMO and for CLUSTAL-Ω are shown in red and green, respectively.

Unlike most MSA programs, GISMO is stochastic and therefore will return a different MSA for each run. This raises the question of GISMO’s run-to-run SP-score variability, as well as how this compares to the variability in SP-scores among distinct deterministic programs. To start, **Fig 3A** plots the range of SP-scores, computed for all of the 408 CDD MSAs, and sorted from lowest to highest values separately for each program. Note that there are many low SP-scores corresponding to sequence sets that are particularly difficult to align correctly. Consistent with the **Fig 2**analyses, GISMO SP-scores are comparatively higher for its more challenging CDD benchmark sets. **Fig 3B** illustrates the run-to-run variability in GISMO's SP-scores, and thus in the alignments it produced. Some may find this variability disturbing, in contrast to the consistent results returned by deterministic programs. However, the consistency of results does not imply reliability. In **Fig 3C and 3D**we plot the range of SP-scores produced by the six programs we have analyzed. The independent variable in these figures corresponds to a position within an array of the 408 CDD test sets; in **Fig 3C** this array is ordered by the (single-run) GISMO SP-score and in **Fig 3D** by the CLUSTAL-Ω SP-score. Comparing these graphs to **Fig 3B**, we see that the results produced by a collection of widely-used programs are considerably more variable than those produced by separate GISMO runs. Also, in **Fig 3C**, it is evident that the SP-scores for other programs are more frequently smaller rather than greater than GISMO's SP-scores. The variability of GISMO's results reflects the inherent uncertainty present in constructing alignments for most real sequence sets, and may provide a sense of the degree of this uncertainly.

### Program runtimes

**Fig 4:**
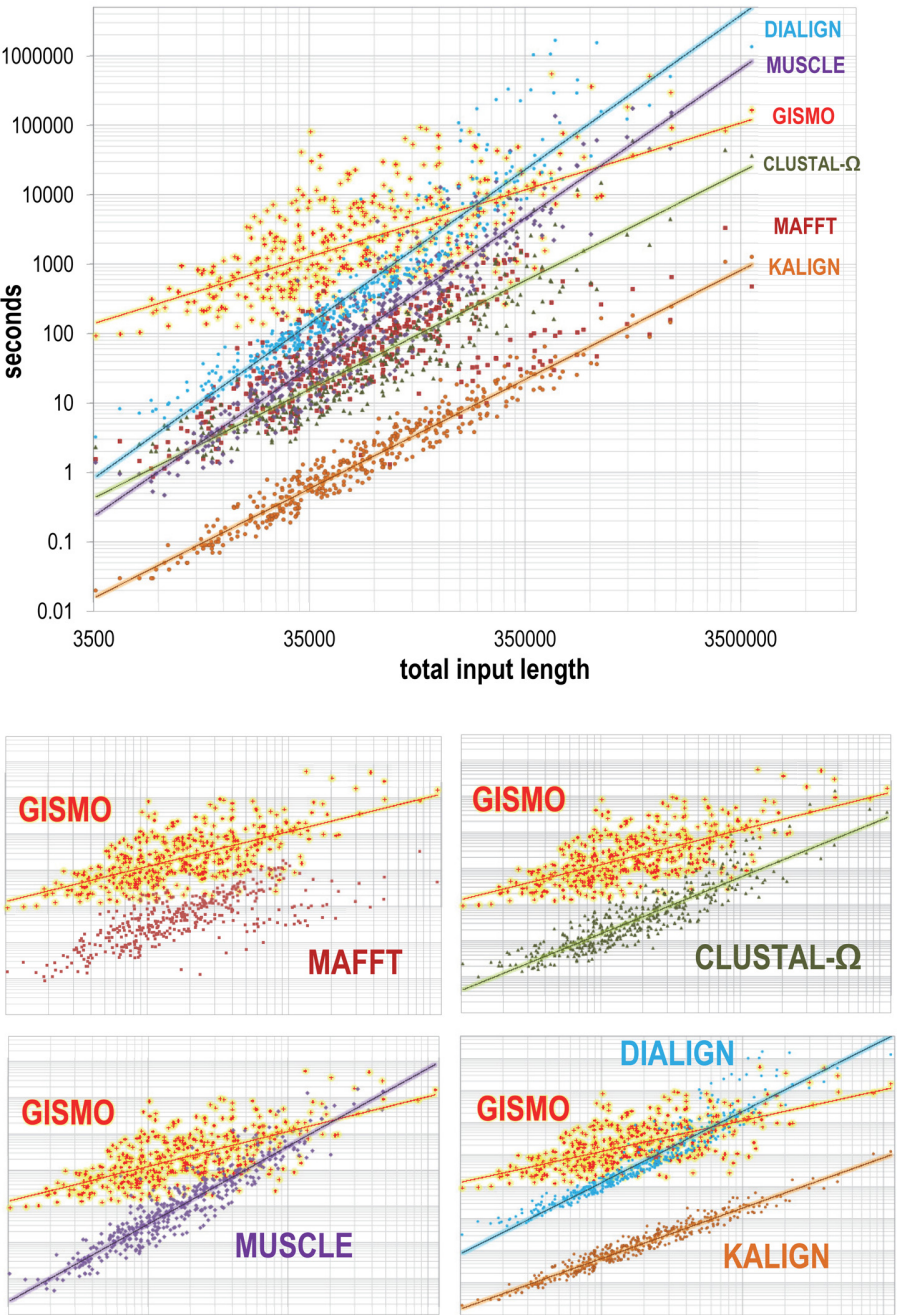
Log-log plots of program runtimes as a function of the total input length. Each data point corresponds to one MSA generated by the program indicated. Estimated time complexities based on trendline slopes were for: GISMO, *t* ∝ *N*^0.96^; Clustal-Ω, *t* ∝ *N*^1.6^; Kalign, *t* ∝ *N*^1.6^; MUSCLE, *t* ∝ *N*^2.1^ and Dialign *t* ∝ *N*^2.2^, where *N* is the total number of residues in the aligned sequences. A trendline is not shown for MAFFT because (with the–auto option) it uses one of several different algorithms depending on the input sequence set; this produces a discontinuity in the data points.

Log-log plots of each program’s runtimes for each of the 408 CDD test sets are given in **Fig 4**, which shows runtimes as a function of the sum of the input lengths for each test set. The average runtime for GISMO was 204 minutes, which is the slowest. GISMO took 971 and 89 times longer to run, on average, than the two fastest programs Kalign and MAFFT, respectively; GISMO took 13 and 4 times longer, on average, than Clustal-Ω and MUSCLE, respectively. However, GISMO’s runtime *t* is roughly estimated to be a linear function of *N* ≡ the total input length based on the slopes of the trendlines in **Fig 4**. The MAFFT data points lack a trendline because MAFFT applies one of several different algorithmic strategies based on the input set, which led to the discontinuity evident in Fig 4. The trendlines for the remaining programs indicate runtimes roughly proportional to *N*^1.6^ and *N*^2.2^. There is considerable variability in GISMO runtimes for a given *N*, presumably due to differences in alignment difficulty: the subtlety and length of conserved regions can vary substantially between test sets having the same *N*. GISMO continues to perform sampling iterations as long as the evolving alignment continues to improve significantly based on its log-likelihood (see Methods). This potential increase in sampling time may be offset by an increase in statistical power with increasing numbers of sequences, thereby allowing the sampler to converge more rapidly due to a higher signal-to-noise ratio. This can explain why the trendline for GISMO runtimes as a function of *N* remains roughly linear. Runtimes for most of the other programs also exhibit a fair amount of scatter for a given *N* (**Fig 4**), but their alignment quality fails to improve with increasing *N* relative to GISMO. GISMO's slowly increasing runtimes and its enhanced alignment quality relative to that of other programs for the progressively larger data sets examined here (**Fig 2**) places it among the methods of choice for even larger data sets consisting of tens of thousands of sequences.

### Prefab benchmarking

Because GISMO is designed to align only those regions conserved by all of the sequences included in the input set, it is most appropriate to benchmark it against CDD alignments, which likewise only align the common conserved region. However, we were curious to know how it performs against a benchmark set designed for MSA programs that globally align all of the input sequences. For this we selected the Prefab benchmark set [31], which consists of 1,682 pairs of structurally aligned sequences. To enlarge each Prefab input set we added up to 1,000 representative homologous sequences (based on how many were available for each family); these expanded Prefab sets (collectively termed Prefab+) are available from the GISMO website. Inasmuch as GISMO will leave unaligned those regions within each Prefab pair that are not conserved in the other sequences, it is disadvantaged relative to the MAFFT, MUSCLE, Clustal-Ω and Kalign programs, which will globally align the input sequences. This is especially true for closely related Prefab sequence pairs, as is illustrated in S1 Text. Despite this handicap, GISMO scores about as well as these other programs on the 1,682 Prefab+ alignments overall (see Wilcoxon signed rank test results in S2 Statistics). Consistent with our CDD benchmark test results, GISMO performs significantly better than these programs on the 841 largest Prefab+ input sets and on the 841 most distantly related Prefab+ input sets; significantly worse on the 841 smallest sets; and significantly worse than MAFFT and Kalign and about the same as MUSCLE and Clustal-Ω on the 841 most similar sequence sets.

### GISMO example alignments

**Fig 5:**
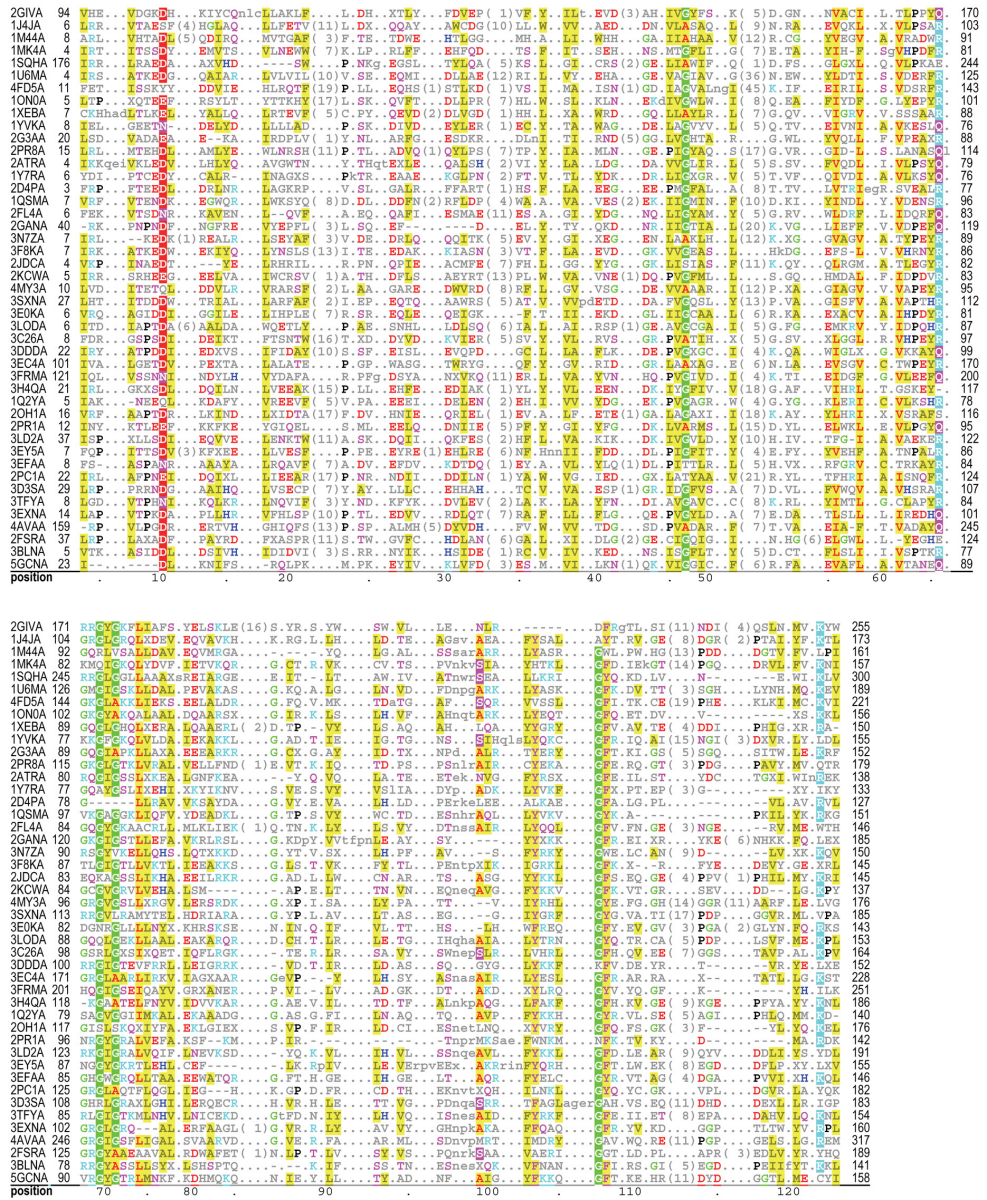
GISMO acetylase domain alignment. Representative proteins of known structure are shown—no two of which share more than 27% sequence identity over the domain footprint. The full alignment consists of 2,125 sequences.

An example of how GISMO aligns representative proteins of known structure for acetylase domain proteins is shown in **Fig 5**. This illustrates how GISMO’s inferred position-specific gap penalties tend to align sequences as conserved indel-sparse “blocks”, which typically correspond to the proteins’ structural core. In contrast, alignments generated by other programs typically have more gaps. This is seen, for example, in **S2–S7 Figs**, which compare the GISMO and MAFFT alignments for representative proteins containing PH, α,β-hydrolase fold and SH2 domains. We chose MAFFT for comparison because it obtained the best GISMO ΔSP-scores when aligning conserved regions within much longer sequences (leftmost data points in **Fig 2C**).

## Discussion

By eliminating the need for a guide tree, our earlier MSA Gibbs samplers [10, 11] constituted a fundamental shift away from conventional progressive alignment methods. They also placed the multiple alignment problem on a firm statistical foundation. However, these samplers modeled indels and amino acid residue prior probabilities inadequately, and often became trapped in misaligned states due to sequence correlations. As a result these samplers were slow and often converged on alignments that were far from optimal. Here we address these inadequacies by incorporating adaptive, position-specific gap penalties, Dirichlet mixture priors and correlated-sequence sampling strategies. As implemented in the GISMO program and illustrated here, these enhancements have yielded improvements in alignment quality.

GISMO’s advantage over progressive alignment methods is most noticeable when a shared domain is present within long, multi-domain proteins. Consider, for example, the MAFFT and GISMO MSAs in **S2 and S3 Figs**, which consist of 582 PH domain protein sequences ranging in length from 94 to 7968 residues with an average length of 891 residues. By iteratively aligning each sequence to a relatively short HMM (the PH domain model consists of about 100 residues) GISMO avoids the problem of aligning pairs of long sequences, consisting mostly of unrelated regions. Moreover, it appears that these unrelated regions can easily mislead progressive alignment methods: Other than GISMO, all of the programs analyzed here will readily align even random sequences. A MAFFT alignment of shuffled PH protein sequences is shown in **S8 Fig**. Clustal-Ω, MUSCLE, Dalign and Kalign likewise will align these same random sequences, although quite differently from MAFFT.

In contrast, due to its statistical basis, GISMO will align only sequences sharing significant similarity. This feature also allows GISMO to identify the HMM architecture and parameters most likely to generate the input sequences and to thereby define the extent of the core alignment more precisely. Sampling from the Bayesian posterior probability distribution leads to output alignment variability, which some biologists might find troubling. However, we believe that this allows a more realistic assessment of what can reliably be inferred from the input sequences than does repeatedly returning the same suboptimal alignment. As illustrated in **Fig 3**, independent sampling runs can also provide some sense of alignment uncertainty.

For some domains, different runs of the current version of GISMO (or runs of different programs) generate significantly differing alignments, some of which appear to be far from optimal. However, the statistical and algorithmic foundations laid here provide avenues for further improvement: Close examination of misaligned regions can suggest new sampling strategies for escaping suboptimal traps. Such strategies may yield more than merely incremental improvement to alignment results. With this in view, we anticipate many further enhancements to GISMO. In particular, there is a large body of literature on MCMC sampling strategies [32] that, when applied, could lead to more rapid convergence on near optimal alignments. More generalized sampling strategies could allow the alignment of multiple copies of a conserved domain within individual sequences, or the automated exclusion of input sequences lacking the conserved domain. Thus far, we have not focused on optimizing GISMO’s code, which lends itself readily to parallelization, and we anticipate being able to increase its speed substantially.

Finally, we ask: What is the benefit of a large, high quality alignment of evolutionarily-related sequences? We suggest an answer through an analogy to physical chemistry: Statistical thermodynamics describes the macroscopic properties of matter as average molecular properties arising from probability distributions over quantum mechanical states. Its central concept is the Boltzmann distribution, which specifies the most probable population of molecular states for a system in thermodynamic equilibrium. This distribution defines all of the thermodynamic properties central to our understanding of chemistry—such as entropy, heat capacity, enthalpy and free energy.

Likewise, the biological properties of proteins may be better understood by considering average properties implied by probability distributions over polypeptide states, with the central concept being a distribution specifying the most probable population of sequences for a protein class in evolutionary equilibrium. GISMO can be used, in combination with a companion MCMC sampler for protein classification [33, 34], to define such a distribution and, by implication, the sequences that arise due to their underlying biochemical properties. Thus, by analogy to thermodynamics, identifying the most statistically striking features of protein sequences indirectly elucidates these biological properties. With this as partial motivation, the GISMO program is being incorporated into a broader project for modeling protein domains [35].

## Methods

### Notation and definitions

The following notation is used for vectors **v** = (*v*_1_,…,*v*_*n*_)^*T*^ and **w** = (*w*_1_,…,*w*_*n*_)^*T*^: |**v**| = |*v*_1_|+…+|*v*_*n*_|, **v**+**w** = (*v*_1_+*w*_1_,…,*v*_*n*_+*w*_*n*_)^*T*^, **v**/**w** = (*v*_1_/*w*_1_,…,*v*_*n*_/*w*_*n*_)^*T*^, $v^{w}=v_{1}{}^{w_{1}}\ldots v_{n}{}^{w_{n}}$, and Γ(**v**) = Γ(*v*_1_)…Γ(*v*_*n*_). Given *K* proteins, their sequences are defined by $R={\left(R_{1}^{T},\ldots ,R_{K}^{T}\right)}^{T}$ where each vector $R_{k}=\left(r_{k,1},\ldots ,r_{k,n_{k}}\right)$ corresponds to the *k*-th sequence, *n*_*k*_ is the *k*-th sequence’s length and the *r*_*k*,*i*_ corresponds to the *i*-th residue in that sequence. **h**( ) defines a counting function where, for example, **h**(*R*_*k*_) returns a length 20 vector of the counts for the residue types in *R*_*k*_.

A block-based alignment of the input sequences is defined by *w* columns. The set of variables defining the sequence positions for column *j* is defined by *A*_*j*_ = {*a*_1,*j*_,…,*a*_*K*,*j*_}. We define *A*_*j*[−*k*]_ ≡ *A*_*j*_ − {*a*_*k*,*j*_} to denote the set *A*_*j*_ without *a*_*k*,*j*_. An alignment is defined by the matrix **A** = (*A*_1_,…,*A*_*w*_)^*T*^ and {**A**} ≡ {*a*_*k*,*j*_: *k* = 1,..,*K*, *j* = 1,…,*w*} denotes the set of residues indices for the alignment variable **A**. We represent the collection of residues indexed by elements in a set C as **R**_*C*_. For instance, **R**_{**A**}_ = {*a*_*k*,*j*_: *k* = 1,…,*K*; *j* = 1,…*w*} represents the set of residues in the alignment defined by **A**.

### GISMO statistical model

The residue frequencies observed for column *c* are modeled as a multinomial distribution with parameters **θ**_*c*_ = (*θ*_1,*c*_,…,*θ*_20,*c*_)^*T*^ where $\sum_{i=1}^{20}\theta_{i,c}=1$ and *θ*_*i*,*c*_ > 0 for all *i*. That is, the vector **Θ** = (**θ**_1_,…,**θ**_*w*_) defines a product multinomial model corresponding to the full alignment. The vector **θ**_0_ corresponds to a background amino acid residue distribution. Hence, the complete-data likelihood function is given by

$$\pi \left(R|\theta_{0},\Theta ,A\right)\propto \theta_{0}^{h\left(R\right)}\prod_{j=1}^{w}{\left(\frac{\theta_{j}}{\theta_{0}}\right)}^{h\left(R_{\left\{A_{j}\right\}}\right)}$$

where it is assumed that Θ ∼ *D*(B) and **θ**_0_ ∼ *D*(**α**) (where *D* denotes the Dirichlet distribution), and where B = (*β*_1_,…,*β*_*w*_) specifies the Dirichlet distribution parameters (commonly interpreted as numbers of pseudocounts) at each column position *j*, and **α** specifies the parameters for the background distribution. (Recall that the alignment is specified by the matrix **A** = (*A*_1_,…,*A*_*w*_) = (*a*_*k*,*j*_)_*K*×*w*_ where *a*_*k*,*j*_ indicates the position of the *j-*th column, which is assumed to be present in all of the sequences.) The likelihood of **A** with the **θ**’s integrated out is

$$\pi \left(R|A\right)\propto \Gamma \left(h\left(R_{{\left\{A\right\}}^{c}}\right)+\alpha \right)\cdot \prod_{j=1}^{w}\Gamma \left\{h\left(R_{\left\{A_{j}\right\}}\right)+\beta_{j}\right\}.$$ (1)

The conditional predictive probability distribution of this conserved region occurring at position *i* in sequence *k* is given by

$$\pi \left(a_{k}=i|A_{\left[-k\right]},R\right)\propto {\prod_{j=1}^{w}\left(\frac{{\hat{\theta }}_{j}}{{\hat{\theta }}_{0}}\right)}^{h\left(r_{k,a_{k,j}}\right)}$$

where the $\hat{\theta }$ are the posterior means of the **θ**, given the observed sequence data **R** and the current alignment **A**_[−*k*]_. This statistical model serves as the foundation for the HMM [10] used in later stages of sampling.

### Dirichlet mixture priors

In order to capture the fact that certain biochemically or structurally similar amino acid residues are more likely to occur together we have incorporated Dirichlet Mixture priors [15, 16], as refined by [17]. In order to speed up sampling, GISMO uses a 20 component mixture in the first (competitive) phase of sample, inasmuch as the goal is to merely obtain a reasonable starting alignment without overtraining the evolving HMM. After this initial phase GISMO applies a 58-component mixture.

### Down weighting for sequence redundancy

Sequences are down weighted for redundancy using the following procedure. For each sequence *k* a non-integer weight is computed using the method of Henikoff and Henikoff [18] as:

$$wt\left(k\right)=\sum_{j=1}^{w}{\left(N_{t_{j}}\cdot N_{r_{k,j}}\right)}^{-1}$$

where $N_{t_{j}}$ is the number of residue types at each position *j* and where $N_{r_{k,j}}=\left|\left\{r_{a_{x,j}}|1\leq x\leq K\wedge r_{a_{x,j}}=r_{a_{k,j}}\right\}\right|$ is the number of sequences with the same residue at position *j* as for sequence *k*. The rationale for this formulation is that if a sequence matches lots of sequences at most positions, then it should receive a lower weight than a sequence that matches few sequences at most positions. These weights are then normalized and integerized as:

$$Wt\left(k\right)=\left\lceil 100\cdot Wt\left(k\right)÷wt_{\text{max}}\right\rceil$$

where *wt*_max_ corresponds to the maximum non-integer sequence weight. Because these weights depend upon the evolving alignment, they are updated after each sampling cycle.

### Inferred HMM transition probabilities

We model the transition probabilities for the HMM shown in **Fig 1B** using a generalization of our previous formulation [10] as follows. The probability matrix for transitions from column *j* states in the HMM is:

$$\begin{array}{cccc} \; & {\text{M}}_{j+1} & {\text{I}}_{j} & {\text{D}}_{j+1}\\ {\text{M}}_{j} & 1-\iota_{o}[j]-\delta_{o}[j] & \iota_{o}[j] & \delta_{o}[j]\\ {\text{I}}_{j} & 1-\iota_{e}[j] & \iota_{e}[j] & 0\\ {\text{D}}_{j} & 1-\delta_{e}[j] & 0 & \delta_{e}[j] \end{array}$$

where 1 ≤ *j* ≤ *w* and where M, I, and D denote match, insertion and deletion states, respectively. The probability matrix for transitions out of the start state is:

$$\begin{array}{ccc} \; & {\text{M}}_{1} & {\text{D}}_{1}\\ \text{Start} & 1-\delta_{o}[0] & \delta_{o}[0] \end{array}$$

Transitions into M and I states emit a residue as specified by the Θ of our statistical model.

*Inference of transition probabilities*. For a given alignment ***A***, each sequence *S_k_* is associated with a “path” through the HMM indicating its alignment against the model Θ. We denote the collection of these paths by Λ and the total number of HMM transitions of type M→M, M→I, …, D→D at position *j* by

$$N_{mm}[j],\;N_{mi}[j],\;N_{md}[j],\;N_{im}[j],\;N_{ii}[j],\;N_{dm}[j]\;\text{and}\;N_{dd}[j].$$

Ignoring the indexing variable *j* for clarity, the likelihood of the transition probability parameters at each position is

$$h(\Lambda |\iota ,\delta )={\left(1-\iota_{o}-\delta_{o}\right)}^{N_{mm}}\iota_{o}^{N_{mi}}\delta_{o}^{N_{md}}{\left(1-\iota_{e}\right)}^{N_{im}}\iota_{e}^{N_{ii}}{\left(1-\delta_{e}\right)}^{N_{dm}}\delta_{e}^{N_{dd}}.$$

with independent prior distributions

$$(\iota_{o},\delta_{o},1-\iota_{o}-\delta_{o})∼D(n_{mi},n_{md},n_{mm}),\;\iota_{e}∼\text{Beta}(n_{ii},n_{im}),\;\text{and}\;\delta_{e}∼\text{Beta}(n_{dd},n_{dm}),$$

where *n*_*mi*_, *n*_*md*_, *n*_*mm*_, *n*_*ii*_, *n*_*im*_, *n*_*dd*_, *n*_*dm*_ are corresponding prior pseudo counts. The corresponding maximum a posteriori probability (MAP) estimates for the transition probabilities at each position *j* are computed from these observed and prior counts. These define the position specific gap penalties. The joint posterior distribution for the alignment and transition probability parameters is

$$g(A,\Lambda ,\vec{\iota },\vec{\delta })\propto P(R|A,\Lambda )\times h(\Lambda |\vec{\iota },\vec{\delta })\times P(\vec{\iota },\vec{\delta }),$$

where *P*(**R**|**A**,Λ) is a generalization of Equation (1), and where $\vec{\iota }$ and $\vec{\delta }$ are length *w* vectors representing the column-specific transition probabilities with prior probability:

$$P(\vec{\iota },\vec{\delta })={\left[D(n_{mi},n_{md},n_{mm})\times \text{Beta}(n_{ii},n_{im})\times \text{Beta}(n_{dd},n_{dm})\right]}^{w}.$$

Given the alignment and thus the paths Λ, we have the conditional posterior distribution

$$\begin{array}{r} p(\vec{\iota },\vec{\delta }|A,\Lambda )\propto \prod_{j=1}^{w}[\iota_{o}{\left[j\right]}^{N_{mi}\left[j\right]+n_{mi}-1}\cdot \delta_{o}{\left[j\right]}^{N_{md}\left[j\right]+n_{md}-1}\cdot {\left(1-\iota_{o}-\delta_{o}\left[j\right]\right)}^{N_{mm}\left[j\right]+n_{mm}-1}\times \\ \iota_{e}{\left[j\right]}^{N_{ii}\left[j\right]+n_{ii}-1}{\left(1-\iota_{e}\left[j\right]\right)}^{N_{im}\left[j\right]+n_{im}-1}\cdot \delta_{e}{\left[j\right]}^{N_{dd}\left[j\right]+n_{dd}-1}{\left(1-\delta_{e}\left[j\right]\right)}^{N_{dm}\left[j\right]+n_{dm}-1}] \end{array}$$

Sampling on the distribution for each position *j* is done by drawing the random variables:

$$\delta_{o}\left[j\right]∼\text{Beta}(N_{md}\left[j\right]+n_{md},N_{mm}\left[j\right]+N_{mi}\left[j\right]+n_{mm}+n_{mi}),$$

$$\delta_{e}\left[j\right]∼\text{Beta}(N_{dd}\left[j\right]+n_{dd},N_{dm}\left[j\right]+n_{dm}),$$

$$\iota_{o}\left[j\right]=(1-\delta_{o}\left[j\right])\iota_{o}^{*}\left[j\right],\;\text{where}\;\iota_{o}^{*}\left[j\right]∼\text{Beta}(N_{mi}\left[j\right]+n_{mi},N_{mm}\left[j\right]+n_{mm}),$$

$$\text{and}\;\iota_{e}\left[j\right]∼\text{Beta}(N_{ii}\left[j\right]+n_{ii},N_{im}\left[j\right]+n_{im}).$$

For computational efficiency, the ι and δ may be integrated out [10] to get

$$\begin{array}{l} h(\Lambda )=\iint h(\Lambda |\vec{\iota },\vec{\delta })P(\vec{\iota },\vec{\delta })d\vec{\iota }d\vec{\delta }\\ \;=\prod_{j=1}^{w}[\frac{\Gamma (N_{mi}\left[j\right]+n_{mi})\Gamma (N_{md}\left[j\right]+n_{md})\Gamma (N_{mm}\left[j\right]+n_{mm})\Gamma (n_{m\cdot })}{\Gamma (N_{m\cdot }\left[j\right]+n_{m\cdot })\Gamma (n_{mi})\Gamma (n_{md})\Gamma (n_{mm})}\\ \;\times \frac{\Gamma (N_{ii}\left[j\right]+n_{ii})\Gamma (N_{im}\left[j\right]+n_{im})\Gamma (n_{i\cdot })}{\Gamma (N_{im}\left[j\right]+N_{ii}\left[j\right]+n_{i\cdot })\Gamma (n_{ii})\Gamma (n_{im})}\\ \;\times \frac{\Gamma (N_{dd}\left[j\right]+n_{dd})\Gamma (N_{dm}\left[j\right]+n_{dm})\Gamma (n_{d\cdot })}{\Gamma (N_{dd}\left[j\right]+N_{dm}\left[j\right]+n_{d\cdot })\Gamma (n_{dd})\Gamma (n_{dm})}]. \end{array}$$

This gives rise to a new posterior distribution *g*(**A**, Λ) ∝ *P*(**R** | **A**, Λ) × *h*(Λ), for which the transition probability parameters need not be fixed or updated and which allows the optimal indel penalties to be determined from the sequence data.

### Sampling algorithm

GISMO’s MCMC sampling algorithm explores the space of possible alignments by executing Markovian transitions between alignments. This involves sampling alternative alignments of either individual sequences or groups of sequences. In either case, such sampling is done as follows: First, the sequence or sequences are removed from the alignment and the posterior parameters of the HMM are recalculated based on the retained aligned sequences and the priors. Next, emission probabilities for the twenty amino acids at each position are sampled from the posterior emission probability distributions defined by the HMM parameters; note that these sampled probabilities define a sampled HMM. Finally, the previously removed sequences are optimally realigned to the sampled HMM. We explored sampling transition probabilities in the same way, but found little benefit of doing so; instead, the MAP estimates for transition probabilities are used. GISMO applies simulated annealing [36] to favor convergence on an optimal alignment in later stages of sampling. Sampling starts at a “temperature” of *T* = 1 (i.e., sample each transition directly proportional to its actual probability *p*) and ends at *T* = 0 (i.e., always take the highest probability transition); between these two extremes the temperature is dropped in *ΔT* = 0.1 increments with sampling probabilities set to p1T. Sampling iteratively through all of the sequences continues until this fails to find a new highest probability state.

### Availability

The GISMO program and the CDD benchmark MSAs and sequence sets used for this study are available at http://gismo.igs.umaryland.edu/.

## Supporting Information

S1 Statistics Excel file with one-tail Wilcoxon signed rank tests for CDD runs. (XLSX)

S2 Statistics Excel file with one-tail Wilcoxon signed rank tests for Prefab+ runs. (XLSX)

S1 Text Explains why Prefab sets are poorly designed for benchmarking GISMO. (PDF)

S1 Charts Excel file containing SP-score data and charts for full length sequence sets. (XLSX)

S2 Charts Excel file containing SP-score data and charts for truncated sequence sets. (XLSX)

S3 Charts Excel file containing SP-score data and charts for multiple GISMO runs. (XLSX)

S1 Runtimes Excel file containing data and charts for program runtime analyses. (XLSX)

S1 Fig Misaligned, correlated sequences within an alignment of enolases. The nine sequences between the two lines are misaligned; the insert residues shown in red correspond structurally to the first 10 columns shown. Note that these misaligned sequences share two distinguishing features: (i) they contain 27–30 residue insertions that the other sequences lack and they conserve a glycine (G) residue in the seventh column instead of the consensus arginine (R) residue. GISMO relies on such features to identify and realign clusters of correlated sequences.
(PDF)

S2 Fig Representative sequences of known structure from a GISMO alignment of 532 PH domains. This corresponds to the same sequences and domain footprint as the MAFFT alignment in S3 Fig.
(PDF)

S3 Fig Representative sequences of known structure from a MAFFT alignment of 582 PH domains. This corresponds to the same sequences and domain footprint as the GISMO alignment in S2 Fig.
(PDF)

S4 Fig Representative sequences of known structure from a GISMO alignment of 836 α,β-hydrolase fold domains. This corresponds to the same sequences and domain footprint as the MAFFT alignment in S5 Fig.
(PDF)

S5 Fig Representative sequences of known structure from the MAFFT alignment of 836 α,β-hydrolase fold domains. This corresponds to the same sequences and domain footprint as the GISMO alignment in S4 Fig.
(PDF)

S6 Fig Representative sequences of known structure from a GISMO alignment of 2,193 SH2 domains. This corresponds to the same sequences and domain footprint as the MAFFT alignment in S7 Fig.
(PDF)

S7 Fig Representative sequences of known structure from the MAFFT alignment of 2,193 SH2 domains. This corresponds to the same sequences and domain footprint as the GISMO alignment in S6 Fig.
(PDF)

S8 Fig MAFFT alignment of 99 randomly shuffled PH domain proteins. (PDF)
