# Supplementary material for: Bayesian Top-Down Protein Sequence Alignment with Inferred Position-Specific Gap Penalties
Source: PLoS Comput Biol. 2016 May 18;12(5):e1004936. doi: 10.1371/journal.pcbi.1004936 (PMC4871425; doi:10.1371/journal.pcbi.1004936)
Supplement: S1 Text — (PDF) [file pcbi.1004936.s003.pdf]

Bayesian Top-Down Protein Sequence Alignment with Inferred Position-Specific Gap Penalties

Prefab benchmarking

Andrew F. Neuwald and Stephen F. Altschul

GISMO is designed to align only those regions conserved by all of the sequences included in the input set. Consequently, it will leave unaligned those regions in each PREFAB sequence pair that are not conserved in the other sequences and hence it is disadvantaged relative to the MAFFT, MUSCLE, Clustal-Ω and Kalign programs, each of which globally aligns the input sequences. This is especially true for closely related Prefab pairs, such as 1d2tA and 1eoiA, the alignment of which is shown here:

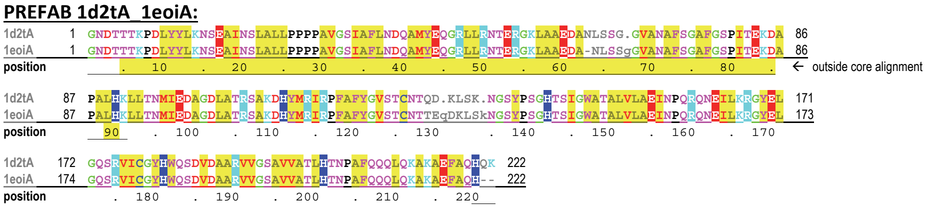

These sequences share about 95% identity over this alignment, whereas sequences in the enlarged set of 263 sequences share much less similarity over the Prefab aligned region, as shown here:

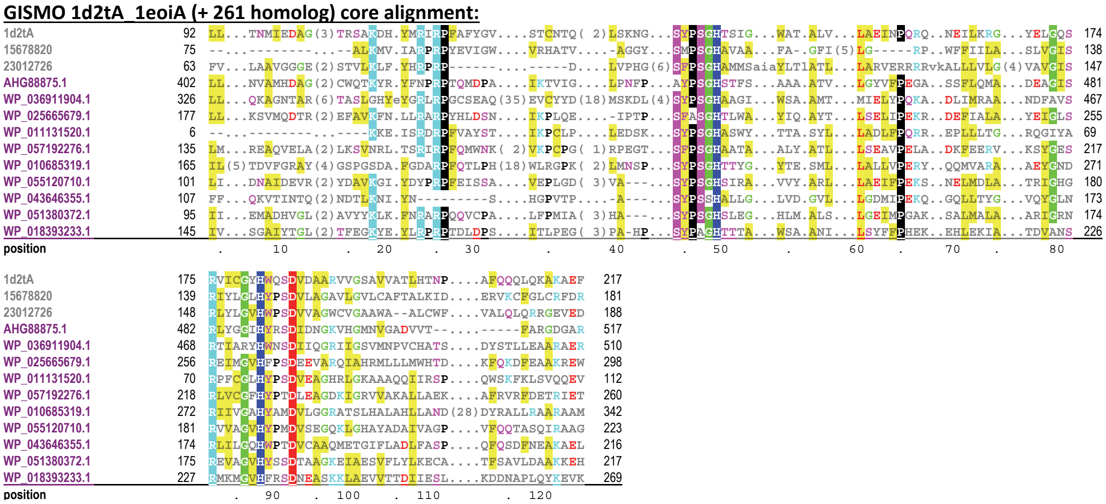

Note that the Prefab 91 residue N-terminal aligned region is not conserved. Despite this handicap, GISMO scores about as well as these other programs on the 1,682 Prefab alignments overall and significantly better on the largest and on the most diverse Prefab+ input sequence sets (see tables below; for full data sets and Wilcoxon test results see S2_stat.xls).

Wilcoxon signed-rank test: all 1682 sets			
GISMO-vs:	average	Z-score	P-value
MAFFT	-0.0167	-1.68	0.05
MUSCLE	0.0151	4.50	$< 10^{-5}$
CLUSTAL-O	0.00480	0.633	0.26
K-align	-0.0191	-3.04	0.001

Wilcoxon signed-rank test: 841 largest sets			
GISMO-vs:	average	Z-score	P-value
MAFFT	0.0377	7.05	$< 10^{-5}$
MUSCLE	0.833	12.0	$< 10^{-5}$
CLUSTAL-O	0.0575	7.70	$< 10^{-5}$
K-align	0.00593	2.23	0.01

Wilcoxon signed-rank test: 841 most diverse sets			
GISMO-vs:	average	Z-score	P-value
MAFFT	0.0103	1.83	0.03
MUSCLE	0.404	4.87	$< 10^{-5}$
CLUSTAL-O	0.0206	1.69	0.05
K-align	0.0282	4.07	2×10^{-5}
