# Supplementary material for: Bayesian Top-Down Protein Sequence Alignment with Inferred Position-Specific Gap Penalties
Source: PLoS Comput Biol. 2016 May 18;12(5):e1004936. doi: 10.1371/journal.pcbi.1004936 (PMC4871425; doi:10.1371/journal.pcbi.1004936)
Supplement: S1 Fig — The nine sequences between the two lines are misaligned; the insert residues shown in red correspond structurally to the first 10 columns shown. Note that these misaligned sequences share two distinguishing features: (i) they contain 27–30 residue insertions that the other sequences lack and they conserve a glycine (G) residue in the seventh column instead of the consensus arginine (R) residue. GISMO relies on such features to identify and realign clusters of correlated sequences. (PDF) [file pcbi.1004936.s008.pdf]

```
2HNE|A  217 IKL......KVGanvqddi.........................................RRCRLARAAIg.P.D.IA..............................M.AV.DAN..QRW... 253
3RR1|A  144 FKL......NGCeemgiidtsravdaav................................ARVAEIRSAFg.N.T.VE..............................F.GL.DFH..GRV... 189
2DW6|A  181 VKM......AIGgapieedr........................................MRIEAVLEEIg.K.D.AQ..............................L.AV.DAN..GRF... 218
3SSZ|A  199 YKM......RFGwgpkdgmdgmrenl..................................KRVEAVREVIg.Y.D.VD..............................L.ML.ECY..MGW... 242
2GSH|A  189 GKMpt....HWGphdgdagirkda....................................AMVADMREKCg.P.D.FW..............................L.ML.DCW..MSQ... 232
4E4U|A  163 VKF......DPAgpytaysghqlslevldrce............................LFCRRVREAVg.S.K.AD..............................L.LF.GTH..GQM... 212
4E5T|A  170 VKF......DPAgaytiydghqpsledlerse............................AFCKQIRAAVg.T.K.AD..............................L.LF.GTH..GQF... 219
4E4F|A  168 IRV......QCGvpgmettygmakgkglayepatkgslpeeqlwstekyldftp......KLFEAVRDKFg.F.N.EH..............................L.LH.DMH..HRL... 239
4FI4|A  169 IRL......QSGvpglpstygvsgdkmfyepadgnlptenvwstskylkhap........KLFEAAREALg.D.D.VH..............................L.LH.DVH..HRL... 238
4F4R|A  169 VRV......QSGvpgiettygvaktpgeryepadsslpaehvwstekylnhap.......KLFAAVRERFg.D.D.LH..............................V.LH.DVH..HRL... 239
4HCH|A  188 IKL......HTWmppvswapdvkmdl..................................KACAAVREAVg.P.D.IR..............................L.MI.DAF..HWY... 231
4GFI|A  149 LKI......KTGtadde...........................................ARLRAVRAAA..P.E.AR..............................I.II.DAN..EGW... 182
4GIR|A  173 IRC......QLGfyggvpeniqtaqnptqgsyydqdqyientv.................EMFKNLREKYg.K.Q.FH..............................I.LH.DVH..ERL... 233
4G8T|A  222 FKL......KGGvldgfeea........................................EAVTALAKRF..P.D.AR..............................I.TL.DPN..GAW... 258
4GYP|C  215 FKL......KGGvlpgeqei........................................DTVRALKKRF..P.D.AR..............................I.TV.DPN..GAW... 251
3UCC|A  191 LKG......VIKdkyg............................................KDATNVGDEGg.F.A.PNilensealelvkeaidkagytekivig...M.DV.AAS..EFY... 251
1E9I|A  192 LAK......VLKak..............................................GMNTAVGDEGg.Y.A.PNlgsnaealaviaeavkaagyelgkditla.M.DC.AAS..EFY... 252
4EWJ|A  189 LKK......ILKar..............................................GLVTAVGDEGg.F.A.PKfegtedgvetiieaieaagyeagengimigF.DC.ASS..EFYdke 253
1ELS|A  193 LKS......LTKkryg............................................ASAGNVGDEGg.V.A.PNiqtaeealdlivdaikaaghdgkvkig...L.DC.ASS..EFF... 253
4G7F|A  190 LKS......IIKkkyg............................................QDAVNVGDEGg.F.A.PPitdineplpilmeaieqaghkgrfaic...M.DS.AAS..ETY... 250
2PTW|A  193 LRG......IIKkkyg............................................QDAVNVGDEGg.F.A.PPikdineplpilmeaieeaghrgkfaic...M.DC.AAS..ETY... 253
3OTR|A  199 LKN......VIKnkyg............................................LDATNVGDEGg.F.A.PNvataeealnllveaikaagyegkikia...F.DA.AAS..EFYkq. 261
3QTP|A  195 LKV......VIKakyg............................................QDATNVGDEGg.F.A.PNvsgarealdllveaiakagytgkieia...M.DC.AAS..EFY... 255
3UJ2|A  213 LAG......LLKsk..............................................GLATSVGDEGg.F.A.PDlasdeeaieyileavklagyepgrdfvla.M.DA.ASS..E-W... 272
1WUE|A  180 VKL......KIRpgydv...........................................EPVALIRQHF..P.N.LP..............................L.MV.DAN..SAY... 213
2ZC8|A  160 IKL......KIKpgwdy...........................................EVLKAVREAF..P.E.AT..............................L.TA.DAN..SAY... 193
1WUF|A  180 VKL......KIApnkdi...........................................QFVEAVRKSF..P.K.LS..............................L.MA.DAN..SAY... 213
1R0M|A  167 IKL......KIKpgwdv...........................................QPVRATREAF..P.D.IR..............................L.TV.DAN..SAY... 200
2MUC|A  166 FKL......KIGanpveqdl........................................KHVVTIKRELg.D.S.AS..............................V.RV.DVN..QYW... 203
1JPM|A  159 LKI......KVGkddiatdi........................................ARIQEIRKRVg.S.A.VK..............................L.RL.DAN..QGW... 196
1MRA|A  163 VKT......KIGypaldqdl........................................AVVRSIRQAVg.D.D.FG..............................I.MV.DYN..QSL... 200
1EC7|A  204 FKL......KGGvlageeea........................................ESIVALAQRF..P.Q.AR..............................I.TL.DPN..GAW... 240
1BQG|A  210 FKL......KGGvlrgedei........................................AAVTALSERF..P.D.AR..............................I.TL.DPN..GAW... 246
4HN8|A  223 FKL......KGGvxrgaeex........................................EAIRAIKARF..P.D.AR..............................V.TL.DPN..GAW... 259
1YEY|A  220 IKL......KVGanvqddi.........................................RRCRLARAAIg.P.D.IA..............................X.AV.DAN..QRW... 256
position               .                                                   90    .                                    100      .
```

Fig. S1. Misaligned, correlated sequences within an alignment of enolases. The nine sequences between the two lines are misaligned; the insert residues shown in red structurally correspond to the first 10 columns shown. Note that these misaligned sequences share two distinguishing features: (i) they contain a 27-30 residue insertions that the other sequences lack and they conserve a glycine (G) residue in the seventh column instead of the consensus arginine (R) residue. GISMO relies on such features to identify and realign clusters of correlated sequences.
