# Supplementary material for: Bayesian Top-Down Protein Sequence Alignment with Inferred Position-Specific Gap Penalties
Source: PLoS Comput Biol. 2016 May 18;12(5):e1004936. doi: 10.1371/journal.pcbi.1004936 (PMC4871425; doi:10.1371/journal.pcbi.1004936)
Supplement: S2 Fig — This corresponds to the same sequences and domain footprint as the MAFFT alignment in S3 Fig. (PDF) [file pcbi.1004936.s009.pdf]

```
2VRW|B  235 KIDGELKITSVer....................RSKTD....R...YAFLLDK........ALLICKRRGDsyd...................LK 275
1V61|A   30 FMSQVVMQHGAc.....................EEKEE....R...YFLLFSS........VLIMLSASPRmsgfm.................YQ 71
1ZC3|B    9 VYNGDLVEYEAdhm...................AQLQR....V...HGFLMND........CLLVATWLPQrrgmyr................YN 53
1KZ7|A  211 LXQGSFSVWTDhkkghtkvkelar.........FKPXQ....R...HLFLHEK........AVLFCKKREEngegyekapsys..........YK 271
1V5U|A    9 SYEGILYKKGAfm....................KPWKA....R...WFVLDKTkh......QLRYYDHRMDte....................CK 50
1MAI|A   14 LKGSQLLKVKS......................SSWRRe...R...FYKLQED........CKTIWQESRKvmrsp.................ES 55
1FOE|A  233 LSXGDLLLHTSviwlnppasl............GKWKKepelA...AFVFKTA........VVLVYKDGSKqkkklvgshrlsiyeewdpfr.FR 303
4K81|A  138 EIHGFLHAKEQgk....................KSWKK....I...YFFLRRS........GLYFSTKGTSaaprhlqffsefgn........SD 189
2COC|A    9 LLCGPLRLSESg.....................ETWSE....V...WAAIPMSdpq.....VLHLQGGSQDgr....................LP 50
2DA0|A   11 EKKGYLLKKSDgir...................KVWQR....R...KCSVKNG........ILTISHATSNr.....................QP 50
3JZY|A  254 LHSGKLYKTKS......................N--KE....L...HGFLFND........FLLLTYMVKQfavssgseklfssksnaqfkm.YK 308
3ULB|A   11 YHKYKVWRRQQmsf...................INKHE....R...TLAIDGD........YIYIVPPEGRihwhdnv...............KT 56
1MKE|A   42 TMSSAVVQLYAadrncmws..............KKCSG....V...ACLVKDNpqrsy...FLRIFDIKDGk.....................LL 91
3DXC|A   32 ALESVLSSSSR......................EQWTP....S...HVSVAPA........TLTILHQQTEa.....................VL 68
2EJ8|A   21 RFLGSMEVKSDdhpdvvyetmrqilaaraihniFRMTE....S...HLLVTCD........CLKLIDPQTQv.....................TR 79
1MI1|A    4 VLSTPAQLIAP......................VVVAK....G...TLSITTT........EIYFEVDEDDsafkkidtkvlayteg......LH 55
2YF0|A    7 TKVEQVKLLDRfsts..................NKSLT....G...TLYLTAT........HLLFIDSHQKet....................WI 48
2DX5|A   12 EINETLVIQQRgvrvydgeek............IKFDA....G...TLLLSTH........RLIWRDQKNNe.....................CC 58
4IFS|A  118 NMSGSLYEMVSrvm...................KALVN....R...KITVPGNfqghsgaqCITCSYKASSgllyplergfiyvhk.......PP 179
4DBB|A   35 QAQEAVSRIKPeges..................QPMTE....V...DLFISTQ........RIKVLNADTQe.....................PM 75
1XCG|A  254 IHEGPLTWRISk.....................DKTLD....L...HVLLLED........LLVLLQKQDEklllkchsktavgssdskqtfsPV 312
1K5D|B   43 EDEEELFKMRAklfrfasendl...........PEWKE....RgtgDVKLLKHkekgai..RLLMRRDKTLki....................CA 100
1M7E|A   37 MSQDSMMKLKGmaaagrsqg.............QHKQR....I...WVNISLS........GIKIIDEKTGv.....................IE 82
1LW3|A  290 PMVGVSGKRSK......................EDEKY....L...QAIMDSNaqsh....KIFIFDARPSvnavankakgggyesedayqnaEL 351
1PFJ|A    4 SSEEVLLIVKKvr....................QKKQD....G...ALYLMAE........RIAWAPEGKDrftish................MY 47
1X1F|A   19 YFEGFLLIKRSgy....................REYEH....Y...WTELRGT........TLFFYTDKKSiiyvd.................KL 61
2ROV|A    2 RLEGWLSLPVRnntkk.................FGWVK....K...YVIVSSK........KILFYDSEQDkeqsn.................PY 47
position        .   10                          .          20            .   30                          .

2VRW|B  276 ASVNLHSFQVRDDSSgerdnkkw......SHM..FLLIEDQga............QGYELFF.KTR..ELKKKWMEQFEMAISNIYP 339
1V61|A   72 GKIPIAGMVVNRLDEiegs..........DCM..FEITGSTv.............ERIVVHC.NNN..QDFQEWMEQLNRLTKSGPS 130
1ZC3|B   54 ALYPLDRLAVVNVKDnppm..........KDM..FKLLMFP..............ESRIFQA.ENA..KIKREWLEVLEETKRALSD 111
1KZ7|A  272 QSLNXTAVGITENVKgd............TKK..FEIWYNAre............EVYIIQA.PTP..EIKAAWVNAIRKVLTSQLQ 329
1V5U|A   51 GVIDLAEVEAVAPGTptigapktvde...KAF..FDVKTTR..............RVYNFCA.QDV..PSAQQWVDRIQSCLSSGPS 115
1MAI|A   56 QLFSIEDIQEVRMGHrteglekfardipeDRC..FSIVFKDqr............NTLDLIA.PSP..ADAQHWVQGLRKIIHHSGS 125
1FOE|A  304 HXIPTEALQVRALPSadaea.........NAV..CEIVHVKsesegrpe......RVFHLCC.SSP..ESRKDFLKSVHSILRDKHR 370
4K81|A  190 IYVSLAGKKKHGAPT..............NYG..FCFKPNKaggpr.........DLKMLCA.EEE..QSRTCWVTAIRLLKYGMQL 248
2COC|A   51 RTIPLPSCKLSVPDPeerlds........GHV..WKLQWAK..............QSWYLSA.SSA..ELQQQWLETLSTAAHSGPS 110
2DA0|A   51 AKLNLLTCQVKPNAEd.............KKS..FDLISHN..............RTYHFQA.EDE..QDYVAWISVLTNSKEEALT 105
3JZY|A  309 TPIFLNEVLVKLPTDpssd..........EPV..FHISHID..............RVYTLRT.DNI..NERTAWVQKIKAASEQYID 366
3ULB|A   57 KSLHISQVVLVKKSKrv............PEH..FKIFVRRegqddi........KRYYFEA.VSG..QECTEIVTRLQNLLSAYRM 118
1MKE|A   92 WEQELYNNFVYNSPR..............GYF..HTFAGDT..............CQVALNF.ANE..EEAKKFRKAVTDLLGRRQR 145
3DXC|A   69 GECRVRFLSFLAVGRd.............VHT..FAFIMAAgpasf.........CCHMFWC.EPN..--AASLSEAVQAACMLRYQ 126
2EJ8|A   80 LTFPLPCVVLYATHQenkr..........LFG..FVLRTSSgrsesnl.......SSVCYIF.ESN..NEGEKICDSVGLAKQIALH 144
1MI1|A   56 GKWXFSEIRAVFSRRyllq..........NTA..LEVFXANr.............TSVXFNF.PDQ..ATVKKVVYSLPRVGVGTSY 114
2YF0|A   49 LHHHIASVEKLALTTs.............GCP..LVIQCKNf.............RTVHFIV.PRE..RDCHDIYNSLLQLSKQAKY 104
2DX5|A   59 MAIPLSQIVFIEEQAagigk.........SAK..IVVHLHPapsnkepgpfqsskNSYIRLS.FKE..HGQIEFYRRLSEEMTQRRW 131
4IFS|A  180 VHIRFDEISFVNFARgttttr........SFD..FEIETKQg.............TQYTFSS.IER..EEYGKLFDFVNAKKLNIKN 240
4DBB|A   76 MDHPLRTISYIADIG..............N--..IVVLMARrrmprsq.......YKMICHV.FES..EDAQLIAQSIGQAFSVAYQ 134
1XCG|A  313 LKLNAVLIRSVATDK..............RAF..FIICTSKlgpp..........QIYELVA.LTS..SDKNTWMELLEEAVRNA-- 368
1K5D|B  101 NHYITPMMELKPNAGs.............DRA..WVWNTHAdfadecpkp.....ELLAIRF.LNA..ENAQKFKTKFEECRKEIEE 164
1M7E|A   83 HEHPVNKISFIARDVtd............NRA..FGYVCGGe.............GQHQFFAiKTG..QQAEPLVVDLKDLFQVIYN 140
1LW3|A  352 VFLDIHNIHVMRESL..............RKL..KEIVYPNie............ETHWL--.-SN..LESTHWLEHIKLILAGALR 404
1PFJ|A   48 ADIKCQKISPEGKAKi.............QLQ..LVLHAGDt.............TNFHFSN.ESTavKERDAVKDLLQQLLPKFKR 105
1X1F|A   62 DIVDLTCLTEQNSTE..............KNCakFTLVLPK..............EEVQLKT.ENT..ESGEEWRGFILTVTELSVP 117
2ROV|A   48 MVLDIDKLFHVRPVTqtdvyradakei..PRI..FQILYANegissa........KNLLLLA.NST..EEQQKWVSRLVKKIPKK-- 117
position      40    .   50                    .   60                  .    70      .   80    .   90
```

Fig. S2. Representative sequences of known structure from a GISMO alignment of 532 PH domains. This corresponds to the same sequences and domain footprint as the MAFFT alignment in Fig. S3.
