# Supplementary material for: Bayesian Top-Down Protein Sequence Alignment with Inferred Position-Specific Gap Penalties
Source: PLoS Comput Biol. 2016 May 18;12(5):e1004936. doi: 10.1371/journal.pcbi.1004936 (PMC4871425; doi:10.1371/journal.pcbi.1004936)
Supplement: S3 Fig — This corresponds to the same sequences and domain footprint as the GISMO alignment in S2 Fig. (PDF) [file pcbi.1004936.s010.pdf]

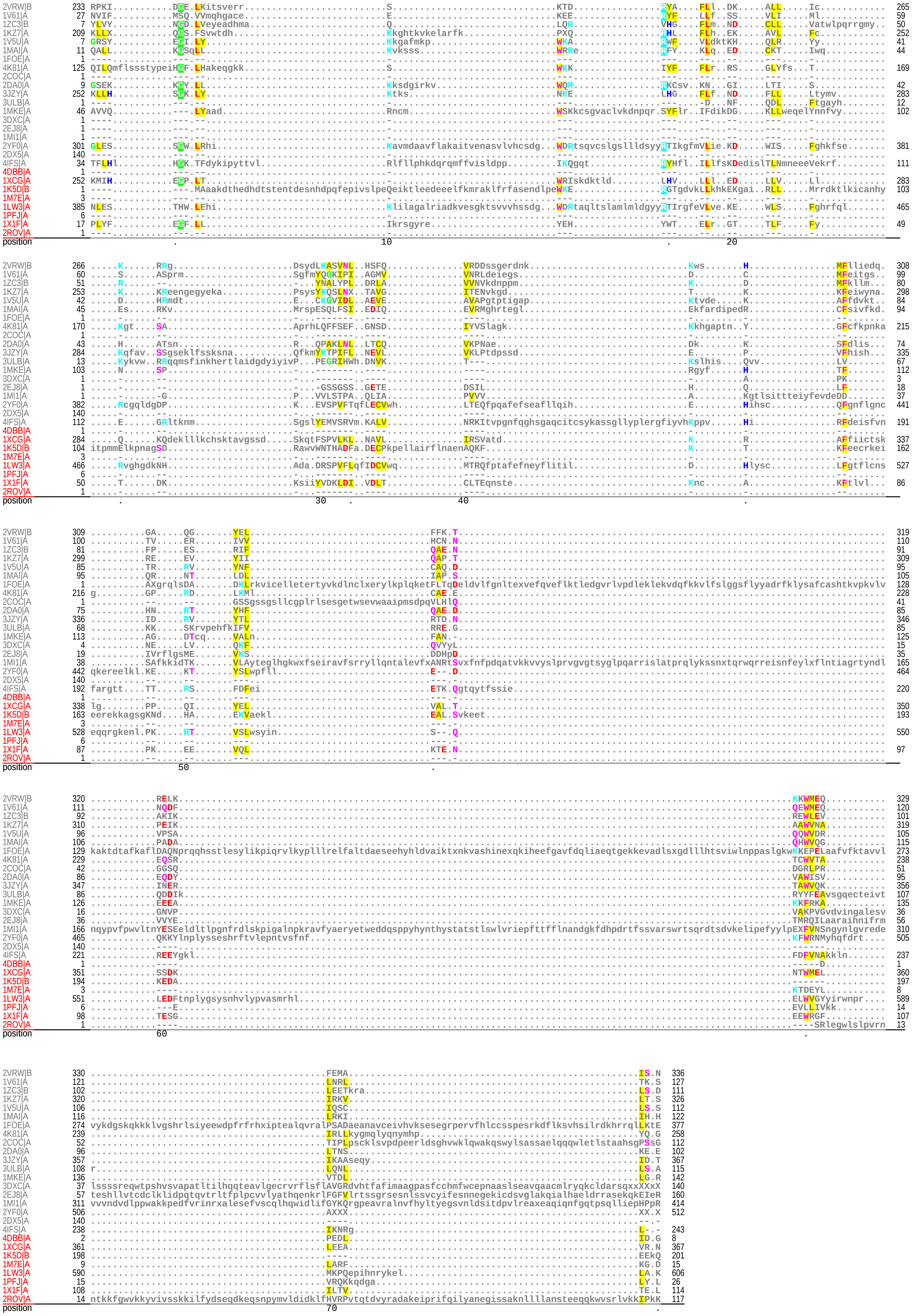

Fig. S3. Representative sequences of known structure from a MAFFT alignment of 582 PH domains. This corresponds to the same sequences and domain footprint as the GISMO alignment in Fig. S2. In order to emphasize the conserved core and to fit the alignment onto a single page, columns containing more than 50% deletions in the complete alignment were removed; these correspond to the insert regions shown here.
