# Supplementary material for: Bayesian Top-Down Protein Sequence Alignment with Inferred Position-Specific Gap Penalties
Source: PLoS Comput Biol. 2016 May 18;12(5):e1004936. doi: 10.1371/journal.pcbi.1004936 (PMC4871425; doi:10.1371/journal.pcbi.1004936)
Supplement: S4 Fig — This corresponds to the same sequences and domain footprint as the MAFFT alignment in S5 Fig. (PDF) [file pcbi.1004936.s011.pdf]

```
1CRL|A   111 GANLPV.....MLWIFGGGfevgGTSTfppaq......................MITKSIAMG......--K....PII...H....VSVNYRvsswG.FLA........G.DEIKAEg.....  173
2ES4|A     5 AATRYP.....VILVHGLA....GTDKfanvvd.....................YWYGIQSDL......QSH....GAK...V....YVANLS....G.FQS........D.DGPNGR......  61
3H2G|A    56 VEGEPAtasg.VLLIPGGE....RCSGpypllgwghptealraqeqakeirdakGDDPLVTRL......ASQ....GYV...V....VGSDYL....G.LGK........SnYAYHPYlhsase  144
3QIT|A    23 SPEHPV.....VLCIHGIL....EQGL...........................AWQEVALPL......AAQ....GYR...V....VAPDLF....G.HGR........S.SHLEMVt.....  74
3C5W|P    39 GSEGPV.....LLLLHGGG....HSAL...........................SWAVFTAAI......ISRv...QCR...I....VALDLR....S.HGE........T.KVKNPE......  90
2PL5|A    43 SSKNNA.....ILICHALS....GDAHaagyhsgsdkkpg..............WWDDYIGPGksf...DTN....QYF...I....ICSNVI....G.GCK........G.SSGPLSihpets  115
3BF8|A    13 QHNNSP.....IVLVHGLF....GSLD...........................NLGVLARDL......-VN....DHN...I....IQVDVR....N.HGL........S.PREPVM......  62
2Y6U|A    44 QRRSRTatrlnLVFLHGSG....MSKV...........................VWEYYLPRLvaad..AEG....NYAidkV....LLIDQV....N.HGD........S.AVRNRGrlgtnf  112
2D5L|A   482 AKKYPV.....IVYVYGGP....HAQLvtk........................TWRSSVGGWdiym..AQK....GYA...V....FTVDSR....G.SAN........R.GAAFEQvihrrl  545
2VTV|A    37 TATKTP.....VIFIHGNG....DNAI...........................SFD--MPPG......NVS....GYG..TparsVYAELK....A.RGYndceifgvT.YLSSSEqgsaqy  103
4G5X|A    21 EGEGAP.....LLMIHGNS....SSGA...........................IFAPQLEGE......IGK....KWR...V....IAPDLP....G.HGK........S.TDAIDPdr....  73
3DDU|A   462 DGSHPA.....FLYGYGGF....NISI...........................TPNYSVSRLifv...RHM....GGI...L....AVANIR....G.GGEy.......G.ETWHKGgilank  522
4OPM|A    41 SSTKPT.....LLLIHGLA....GSRD...........................NWNRVAHYL......-TT....NYH...V....IIPDLP....G.SGE........T.IVSQDF......  90
2QMQ|A    32 KPKRPA.....IFTYHDVG....LNYKs..........................CFQPLFRFG......DMQeiiqNFV...R....VHVDAP....G.MEE........G.APVFPLgy....  89
1HLG|A    47 TGQRPV.....VFLQHGLL....ASAT...........................NWISNLPNNslafilADA....GYD...V....WLGNSR....G.NTWarrnlyy.S.PDSVEFwafsfd  116
1LPA|B    66 KTNRKT.....RFIIHGFIdk..GEEN...........................WLANVCKNLf.....KVE....SVN...C....ICVDWK....G.GSRt.......G.YTQASQni....  122
1AZW|A    31 NPHGKP.....VVMLHGGP....GGGC...........................N-DKMRRFH......DPA....KYR...I....VLFDQR....G.SGR........S.TPHADLv.....  81
3AZP|A   420 ADELPPy....VVMAHGGP....TSRVpa.........................VLDLDVAYF......TSR....GIG...V....ADVNYG....GsTGY........G.RAYRERlrgrwg  480
position           .      10     .                      20    .        30              .   40            .    50

1CRL|A   174 ........SAnaGLKDQRLGMQWVADNiaafgGDP...TKV..T.IFGESAGSMSVMCHILWND...GDntykgkplFRA...GIM...QS......GAMVpsdavd.................  251
2ES4|A    62 ........--..-GEQLLAYVKQVLAA.....TGA...TKV..N.LIGHSQGGLTSRYVAAVAP...QL........VAS...VTT...IG......TPHR.......................  115
3H2G|A   145 ........AS..ATIDAMRAARSVLQH.....LKTplsGKV..M.LSGYSQGGHTAMATQREIE...AH........LSKefhLVA...SA......PISGpyaleqtfldswsgsnavgentf  230
3QIT|A    75 ........SY..SSLTFLAQIDRVIQE.....LPD...QPL..L.LVGHSMGAMLATAIASVRP...KK........IKE...LIL...VE......LPLPaeeskkesavnqlttcldyls..  152
3C5W|P    91 ........DL..SAETMAKDVGNVVEAm....YGDlp.PPI..M.LIGHAMGGAIAVHTASSNLvp.SL........LGL...CMIdvvEG......TAMD.......................  155
2PL5|A   116 tpygsrfpFV..SIQDMVKAQKLLVES.....LGI...EKL..FcVAGGSMGGMQALEWSIAYP...NS........LSN...CIV...MA......STAE.......................  181
3BF8|A    63 ........--..NYPAMAQDLVDTLDA.....QQI...DKA..T.FIGHSMGGKAVMALTALAP...DR........IDK...LVA...ID......IAPVdyhvrr.................  123
2Y6U|A   113 ........NWidGARDVLKIATXELGS.....IDS...HPAlnV.VIGHSMGGFQALACDVLQP...NL........FHL...LIL...IE......PVVItrkaigagrpg............  184
2D5L|A   546 g.......QT..EMADQMCGVDFLKSQsw...VDA...DRI..G.VHGWSYGGFMTTNLMLTHG...DV........FKV...GVA...GG......PVID.......................  605
2VTV|A   104 ........NY..HSSTKYAIIKTFIDKvkay.TGK...SQV..D.IVAHSMG-VSMSLATLQYY...NNwts.....VRK...FIN...LA......GGIRglyscyytgyanaaaptcgsqny  189
4G5X|A    74 ........SY..SMEGYADAMTEVMQQ.....LGI...ADA..V.VFGWSLGGHIGIEMIARYP...EM........-RG...LMI...TG......TPPV.......................  129
3DDU|A   523 ........QN..CFDDFQCAAEYLIKEgy...TSP...KRL..T.INGGSNGGLLVAACANQRP...DL........FGC...VIA...QV......GVMD.......................  581
4OPM|A    91 ........DY..SVPNLAEKLRRFVEA.....ANLk..GPI..H.IAGHSLGGSIALLYAGQYP...FE........TKS...LFL...VD......SGGI.......................  148
2QMQ|A    90 ........QYp.SLDQLADMIPCILQY.....LNF...STI..I.GVGVGAGAYILSRYALNHP...DT........VEG...LVL...INidpnakGWMDwaahk..................  158
1HLG|A   117 ........EM..AKYDLPATIDFIVKK.....TGQ...KQL..H.YVGHSQGTTIGFIAFSTNPslaKR........IKT...FYA...LA......PVATvkytkslinklrfvpqslfkfif  199
1LPA|B   123 ........RI..VGAEVAYFVEFLQSA.....FGYsp.SNV..H.VIGHSLGAHAAGEAGRRTN...GT........IGR...ITG...LD......PAEPcfqgtpelvr.............  191
1AZW|A    82 ........DN..TTWDLVADIERLRTH.....LGV...DRW..Q.VFGGSWGSTLALAYAQTHP...QQ........VTE...LVL...RG......IFLLrrfelewf...............  146
3AZP|A   481 ........VV..DVEDCAAVATALAEEgt...ADR...ARL..A.VRGGAAGGWTAASSLVST-...DV........YAC...GTV...LY......PVLDllgw...................  542
position                 .   60     .        70        .    80   .   90        .              100             .

1CRL|A   301 SL.RL........SYLPRPDGV.NITDDM....YALV.....REG.......KY.AN..IPviigdqn.......DEGTFFGTSSLN.....VTTDAqAREYFKQS  365
2ES4|A   166 TL.TT........AQTATYNRN.FPSAGLgap.GSCQ.....TGA.......AT.ET..VG..............GSQHLLYSWGGT.....AI-QP.TSTVLGVT  224
3H2G|A   326 TP.TL........LCGSSNDAT.VPLKNA....QTAI.....ASFqqrgsn.QV.AL..VDtgtgnas.......DNSAFAHMLTKE.....SCIVV.VRDQLLDK  395
3QIT|A   232 VP.TT........LVYGDSSKL.NRPEDL....QQQK.....MTM.......TQ.AKrvFL..............SGGHNLHIDAAA.....ALASL.ILT----S  286
3C5W|P   238 IP.KL........LLLAGVDRL.DKDLTI....GQMQ.....GKF.......QM.QV..LP..............QCGHAVHEDAPD.....KVAEA.VATFLIRH  294
2PL5|A   301 CR.FL........VVSYSSDWL.YPPAQS....REIV.....KSLeaa....DK.RV..FYvelqs.........GEGHDSFLLKNP.....KQIEI.LKGFLENP  365
3BF8|A   196 HP.AL........FIPGGNSPY.VSEQYR....DDLLaqf..PQA.......RA.HV..IA..............GAGHWVHAEKPD.....AVLRA.IRRYLNDH  255
2Y6U|A   285 KR.TI........HIVGARSNW.XPPQNQlfl.QKTL.....QNY.......HL.DV..IP..............GGSHLVNVEAPD.....LVIER.INHHIHEF  344
2D5L|A   642 GR.LM........LIHGAIDPV.VVWQHS....LLFL.....DACvkartypDY.YV..YP..............SHEHNVMGPDRV.....HLYET.ITRYFTDH  705
2VTV|A   246 CA.TA........SFWAGCDSA.AKFASTtsnvKAQInvgagSNA.......TQ.AD..YD..............WADGMPYNAGGG.....DTTNG.VGHFRTKT  311
4G5X|A   209 LP.IA........VVNGRDEPF.VELDFV....SKVKf....GNLweg....KT.HV..ID..............NAGHAPFREAPA.....EFDAY.LARFIRDC  269
3DDU|A   629 YPsML........LLTADHDDR.VVPLHS....LKFI.....ATL.......QY.IV..GRsrkqnnpllihvdtKAGHGAGKPTAKvieevsDMFAF.IARCLNVD  706
4OPM|A   235 AP.TL........ILWGKQDKI.INVEVAnel.KRLL.....KNA.......QPpVI..LE..............NVGHXPILEAEQ.....LVIQQ.YVPFLLKV  295
2QMQ|A   228 CP.VM........LVVGDQ---.APHEDAv...VECN.....SKLdptqt..SF.LK..MA..............DSGGQPQLTQPG.....KLTEA.FKYFLQG-  286
1HLG|A   306 VP.IA........VWNGGKDLL.ADPQDV....GLLL.....PKLpnli...YH.KE..IP..............FYNHLDFIWAMDapq..EVYND.IVSMISED  369
1LPA|B   240 KN.ILsqivdidgIWEGTRDFAaCNHLRS....YKYY.....TDSivnpd..GF.AG..FP..............CASYNVFTANKCfpcps.GGCPQ.MGHYADRY  315
1AZW|A   256 IP.GV........IVHGRYDVV.CPLQSAwdl.HKAW.....PKA.......QL.QI..SP..............ASGHSAFEPENV.....DA---.LVRATDGF  312
3AZP|A   583 VP.FL........LLQGLEDPV.CPPEQC....DRFL.....EAVagcgvphAY.LS..FE..............GEGHGFRRKETMvr....ALEAE.LSLYAQVF  648
position                    .  160     .        170            .      180                   .  190         .  200     .
```

Fig. S4. Representative sequences of known structure from a GISMO alignment of 836 α,β-hydrolase fold domains. This corresponds to the same sequences and domain footprint as the MAFFT alignment in Fig. S5.
