# Supplementary material for: Bayesian Top-Down Protein Sequence Alignment with Inferred Position-Specific Gap Penalties
Source: PLoS Comput Biol. 2016 May 18;12(5):e1004936. doi: 10.1371/journal.pcbi.1004936 (PMC4871425; doi:10.1371/journal.pcbi.1004936)
Supplement: S6 Fig — This corresponds to the same sequences and domain footprint as the MAFFT alignment in S7 Fig. (PDF) [file pcbi.1004936.s013.pdf]

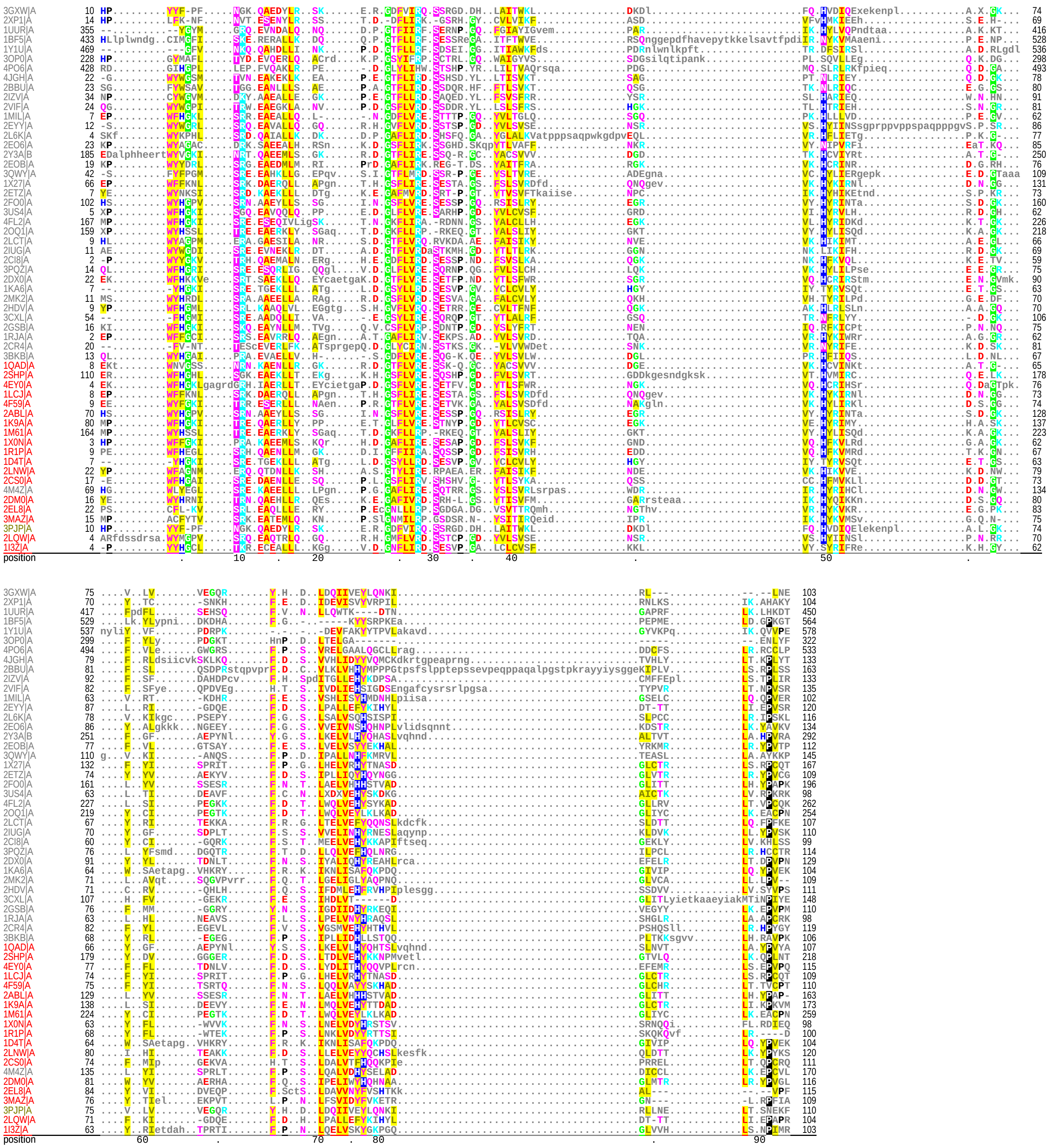

Fig. S6. Representative sequences of known structure from a GISMO alignment of 2,193 SH2 domains. This corresponds to the same sequences and domain footprint as the MAFFT alignment in Fig. S7.
