# Supplementary material for: Bayesian Top-Down Protein Sequence Alignment with Inferred Position-Specific Gap Penalties
Source: PLoS Comput Biol. 2016 May 18;12(5):e1004936. doi: 10.1371/journal.pcbi.1004936 (PMC4871425; doi:10.1371/journal.pcbi.1004936)
Supplement: S7 Fig — This corresponds to the same sequences and domain footprint as the GISMO alignment in S6 Fig. (PDF) [file pcbi.1004936.s014.pdf]

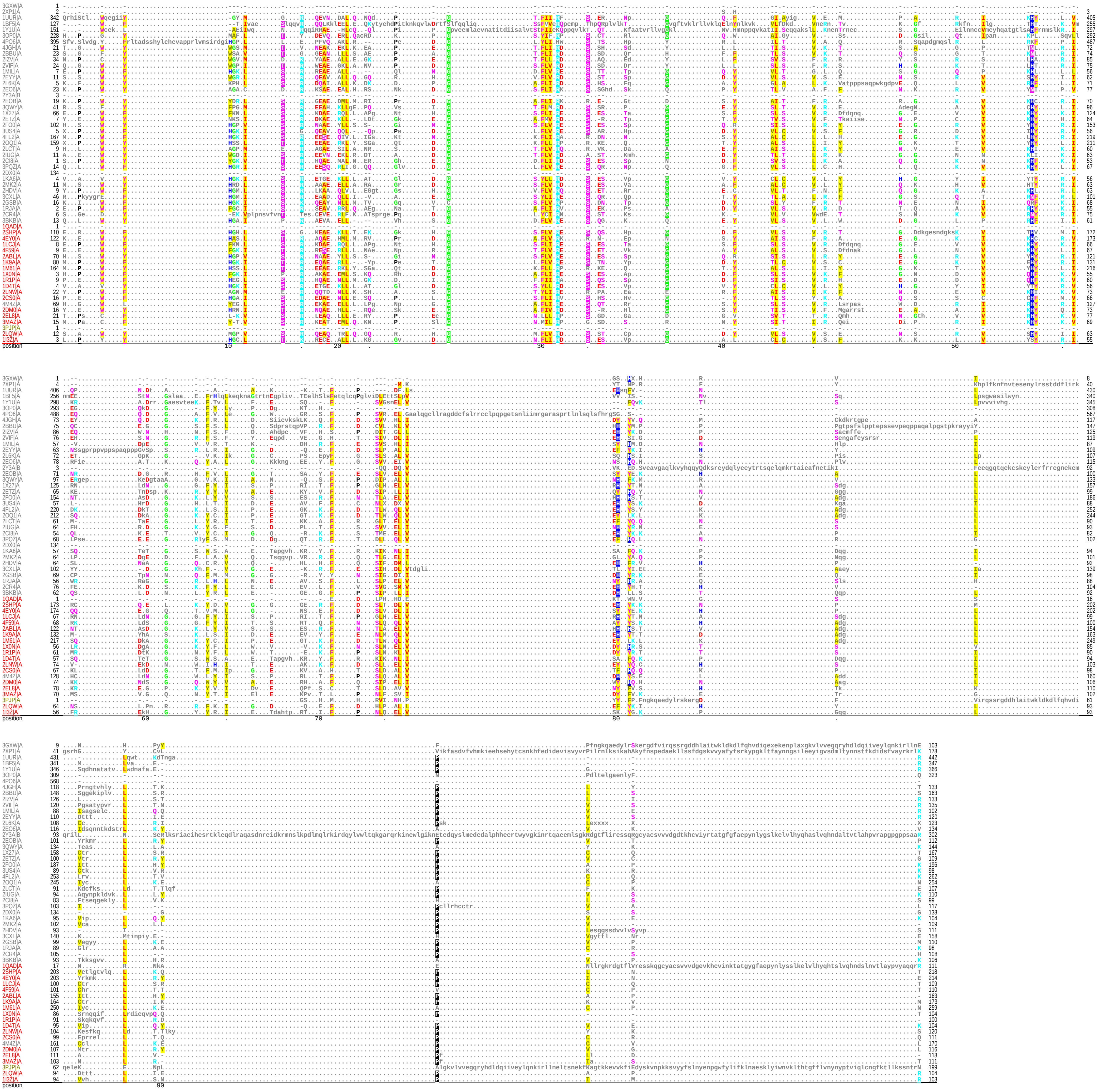

Fig. S7. Representative sequences of known structure from the MAFFT alignment of 2,193 SH2 domains. This corresponds to the same sequences and domain footprint as the GISMO alignment in Fig. S6. In order to emphasize the conserved core, columns containing more than 50% deletions in the complete alignment were removed; these correspond to the insert regions shown here.
