# Supplementary material for: Bayesian Top-Down Protein Sequence Alignment with Inferred Position-Specific Gap Penalties
Source: PLoS Comput Biol. 2016 May 18;12(5):e1004936. doi: 10.1371/journal.pcbi.1004936 (PMC4871425; doi:10.1371/journal.pcbi.1004936)
Supplement: S8 Fig — (PDF) [file pcbi.1004936.s015.pdf]

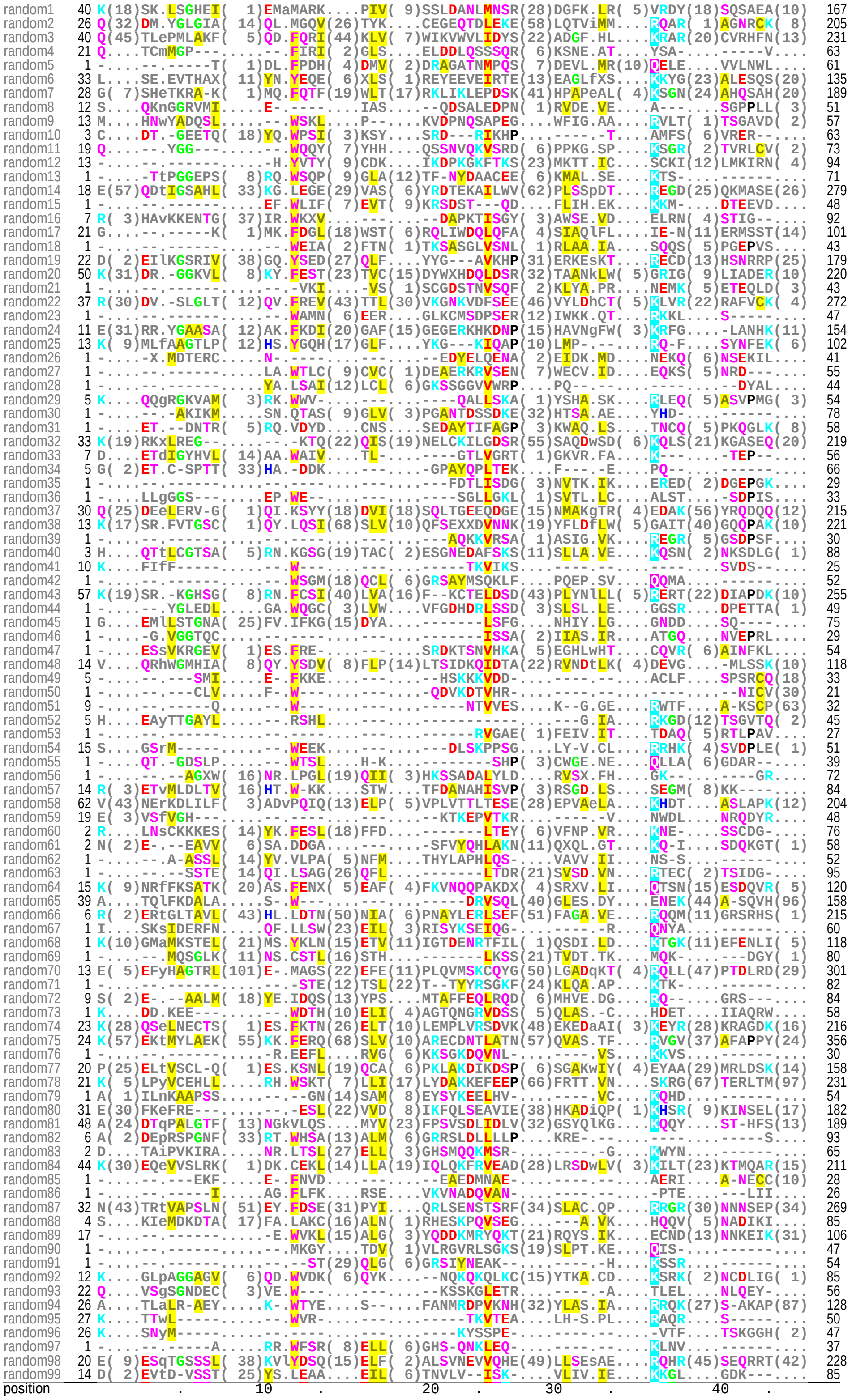

Fig. S8. MAFFT alignment of 99 randomly shuffled PH domain proteins.
